# High-dimensional phenotyping of the peripheral immune response in community-acquired pneumonia

**DOI:** 10.3389/fimmu.2023.1260283

**Published:** 2023-11-24

**Authors:** Tom D. Y. Reijnders, Alex R. Schuurman, Jan Verhoeff, Marlous van den Braber, Renée A. Douma, Daniël R. Faber, Alberta G. A. Paul, W. Joost Wiersinga, Anno Saris, Juan J. Garcia Vallejo, Tom van der Poll

**Affiliations:** ^1^ Center for Experimental and Molecular Medicine (CEMM), Amsterdam UMC location University of Amsterdam, Amsterdam, Netherlands; ^2^ Department of Molecular Cell Biology and Immunology, Amsterdam UMC location Vrije Universiteit Amsterdam, Amsterdam, Netherlands; ^3^ Department of Internal Medicine, Flevo Hospital, Almere, Netherlands; ^4^ Department of Internal Medicine, BovenIJ Hospital, Amsterdam, Netherlands; ^5^ Application Department, Cytek Biosciences, Inc., Fremont, CA, United States; ^6^ Division of Infectious Diseases, Amsterdam UMC location University of Amsterdam, Amsterdam, Netherlands; ^7^ Infectious Disease, Leiden Universitair Medisch Centrum, Leiden, Netherlands

**Keywords:** pneumonia, immunophenotyping, host response, COVID-19, spectral flow cytometry, monocytes, immunosuppression, chemokine receptors

## Abstract

**Background:**

Community-acquired pneumonia (CAP) represents a major health burden worldwide. Dysregulation of the immune response plays an important role in adverse outcomes in patients with CAP.

**Methods:**

We analyzed peripheral blood mononuclear cells by 36-color spectral flow cytometry in adult patients hospitalized for CAP (n=40), matched control subjects (n=31), and patients hospitalized for COVID-19 (n=35).

**Results:**

We identified 86 immune cell metaclusters, 19 of which (22.1%) were differentially abundant in patients with CAP versus matched controls. The most notable differences involved classical monocyte metaclusters, which were more abundant in CAP and displayed phenotypic alterations reminiscent of immunosuppression, increased susceptibility to apoptosis, and enhanced expression of chemokine receptors. Expression profiles on classical monocytes, driven by CCR7 and CXCR5, divided patients with CAP into two clusters with a distinct inflammatory response and disease course. The peripheral immune response in patients with CAP was highly similar to that in patients with COVID-19, but increased CCR7 expression on classical monocytes was only present in CAP.

**Conclusion:**

CAP is associated with profound cellular changes in blood that mainly relate to classical monocytes and largely overlap with the immune response detected in COVID-19.

## Introduction

Community-acquired pneumonia (CAP) is a preeminent driver of hospitalization, short-term mortality and long-term decline (1–4). CAP is initiated by a pathogen, but symptoms and life-threatening complications are primarily caused by the collateral damage of dysregulated host defense mechanisms aimed at eradicating the causative microorganism (1, 4, 5). While *in vitro* and animal studies have generated extensive knowledge of the phenotype and function of immune cells involved in pneumonia pathophysiology, studies that report in-depth cellular phenotyping in patients with CAP remain scarce. In stark contrast, numerous deep immunological profiling studies have been performed in patients with CAP caused by severe acute respiratory syndrome coronavirus 2 (SARS-CoV-2) – the pathogen responsible for coronavirus disease (COVID)-19 – relaying intricate details on immune phenotypes and functional states at single cell level (6). This knowledge has contributed to various immunotherapeutic trials, and the successful application of some of these therapies in clinical practice (7). No such immunotherapies are considered standard of care in patients with other forms of CAP.

We recently reported on the immune response in different forms of CAP based on single-cell RNA sequencing and an extensive set of host response plasma biomarkers (8, 9), and we described differences in lung and blood immunophenotypes in critically ill patients with COVID-19 (10). Here, we use high-dimensional spectral flow cytometry to provide a comprehensive analysis of the peripheral blood immune system compartment at the single-cell protein level in patients with CAP, using age- and sex-matched control subjects without acute infection and patients with COVID-19 as comparator groups.

## Materials and methods

### Study design and participants

This project was part of the ELDER-BIOME study (ClinicalTrials.gov Identifier: NCT02928367) (8, 9). Patients for this project were consecutively enrolled between October 2018 and March 2020 for CAP and in April and May 2020 for COVID-19 (prior to the introduction of the Alpha variant, the availability of vaccines, and dexamethasone becoming standard of care). Inclusion criteria were as reported previously (9), with additional exclusion criteria for comorbidities associated with an immunocompromised state (**Supplemental Methods**). Age- and sex-matched control subjects without signs of acute infection were recruited from the outpatient clinic. The study was approved by the medical ethical committee of the Amsterdam University Medical Centers. All patients or their representatives provided written informed consent.

### Sampling

Blood samples were obtained from patients within 48 hours of admission to a general hospital ward and processed within 4 hours after collection. Peripheral blood mononuclear cells (PBMCs) were isolated from heparin-anticoagulated blood by density gradient centrifugation using Ficoll-Paque plus medium (GE Healthcare Life sciences, Eindhoven, The Netherlands) and cryopreserved in liquid nitrogen until further use, as described (8). Plasma was obtained from EDTA-anticoagulated blood by centrifugation and stored at -80 °C until use.

### Spectral flow cytometry – staining, data acquisition and analysis

We performed 36-color spectral flow cytometry based on the Optimized Multicolor Immunofluorescence Panel (OMIP) 069 (11). Details on experimental procedures and antibodies used can be found in the **Supplemental Methods** and **Supplementary Table 1**. Live single CD45+ cells (**Supplementary Figure 1**) were clustered using FlowSOM (12) (version 1.18) into 600 SOM clusters. The optimal number of metaclusters (MCs) was determined as described in the **Supplemental Methods** and **Supplementary Figure 2A**. MCs were phenotyped by comparison to manual gating strategies as described (11). MCs with the same immunological phenotype were given numbers, and represent differential (activation) states of these phenotypes.

### Protein biomarker assays

Plasma protein biomarkers were measured using Luminex (R&D systems, Minneapolis, Minnesota, USA) or cytometric bead array (BioLegend, San Diego, California, USA) as described (9).

### Statistical analysis

**Figure 1:**
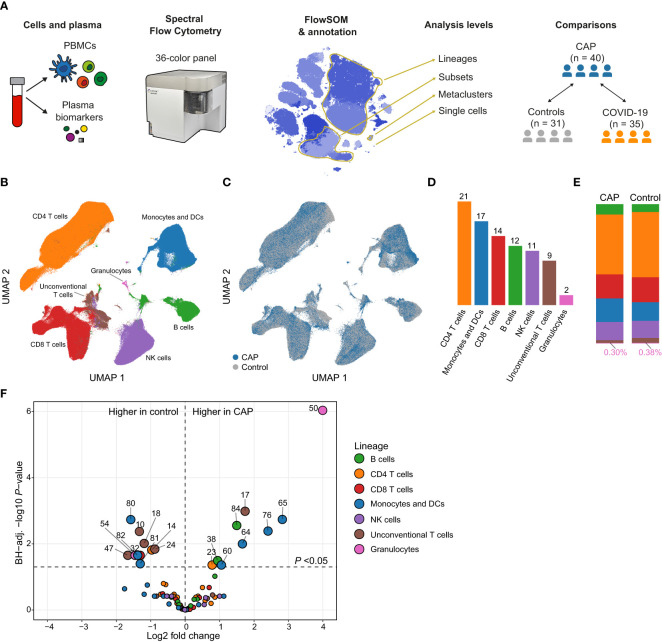
Circulating immune cell frequencies in patients with community-acquired pneumonia (CAP) and controls. **(A)** Experimental design: we obtained peripheral blood mononuclear cells (PBMCs) and plasma from patients with CAP and two comparator groups: controls without signs of acute infection and disease controls with COVID-19. We used 36-color spectral flow cytometry to acquire surface marker expression patterns for each cell and clustered cells using FlowSOM. We performed analyses at several levels (1): individual cells; (2) MCs; (3) cell subsets, defined as all MCs that fall within the same gate as determined by manual gating (e.g. we find nine classical monocyte MCs that altogether make up one classical monocyte subset); and (4) lineages, assigned to MCs based on commonly used groupings in the immunological literature. In some analyses, we compared MCs as whole, whereas in others we look at the values of individual subjects whose cells make up that MC (e.g. the median fluorescence intensity [MFI] of HLA-DR for a subject within an MC). **(B)** Uniform manifold approximation and projection (UMAP) representative of all PBMCs from patients with CAP and controls, colored by lineage (color codes as in **D–F**) or **(C)** by group. **(D)** Number of metaclusters (MCs) per lineage. **(E)** Stacked bar charts indicating the mean of each lineage as a proportion of total cells for patients with CAP and controls. The proportion of (contaminating) granulocytes in the PBMC fraction was very low and therefore indicated in text at the bottom of each bar. **(F)** Volcano plot for the comparison of all 86 MCs (as proportions of total number of cells per subject). The X-axis depicts the difference in means of the log2-transformed proportion of each MC, the Y-axis depicts the -log10-transformed Benjamini-Hochberg- (BH)-adjusted *P*-value obtained using Welch’s *t*-test. Larger labelled points above the horizontal line represent significantly differentially abundant MCs. DC, dendritic cell; NK, natural killer.

We initially determined which MCs were differentially abundant between patient groups in an untargeted manner (i.e. the volcano plot) by performing Welch’s *t*-test on log2-transformed MC proportions (of total cells), and applied Benjamini-Hochberg (BH) adjustment to the resulting *P*-values. In subsequent analyses, we expressed the difference in log2-transformed MC proportions between patient groups and controls by Hedges’ *g*, a commonly used effect size measure that incorporates both the differences in means and a shared measure of variance (13). An overview of the study design, including analysis levels and comparisons, is depicted in **Figure 1A**. Further details can be found in the **Supplemental Methods**.

## Results

### Patient characteristics

We enrolled 40 consecutive patients with CAP within 48 hours after hospital admission, and 31 age- and sex-matched control subjects without signs of acute infection (**Table 1**). Patients and controls were well-matched, although patients more often had chronic obstructive pulmonary disease. Vital signs, disease severity scores and clinical outcomes reflected an overall low to moderate severity of disease; all patients survived up to day 28.

**Table 1:** Baseline characteristics and outcomes.

	CAP (n = 40)	Controls (n = 31)	*P*-value
DEMOGRAPHICS			
Age, years	68.8 (15.4)	64.3 (15.6)	0.23
Sex, male	24 (60.0)	20 (64.5)	0.81
COMORBIDITIES			
Chronic obstructive pulmonary disease	15 (37.5)	2 (6.5)	0.002
Asthma	6 (15.0)	1 (3.2)	0.13
Hypertension	16 (40.0)	15 (48.4)	0.63
Diabetes mellitus, type 2	4 (10.0)	7 (22.6)	0.19
Chronic kidney disease	3 (7.5)	3 (9.7)	>0.99
LABORATORY TESTS*			
Platelets, x10^9^ cells/L	273.9 (119.7)		
Leukocytes, x10^9^ cells/L	13.2 [9.4, 17.0]		
Neutrophils, x10^9^ cells/L	11.5 [7.2, 14.9]		
Lymphocytes, x10^9^ cells/L	0.80 [0.60, 1.28]		
Neutrophil-to-lymphocyte ratio	9.9 [7.3, 15.4]		
C-reactive protein, mg/L	150.8 [68.2, 264.5]		
VITAL SIGNS AND DISEASE SEVERITY*			
Mean arterial pressure, mmHg	95 (14)		
Respiratory rate, bpm	22 [19, 28]		
Temperature, °C	37.8 (1.3)		
Modified Early Warning Score	3 [2, 4]		
Pneumonia Severity Index	4 [2, 4]		
CURB-65	1 [1, 2]		
qSOFA	1 [0, 1]		
CLINICAL COURSE AND OUTCOMES			
Symptoms to admission, days	3 [2, 5]		
Pathogen identified^†^	16 (40.0)		
ICU stay (at any point during admission)	3 (7.5)		
Hospital length of stay, days	5 [3, 9]		
Time to clinical stability^††^ or discharge, days	5 [2, 8]		
28-day mortality	0 (0)		

CAP, community-acquired pneumonia; bpm, breaths/beats per minute; CURB-65, confusion, blood urea nitrogen, respiratory rate, blood pressure, age 65 or older; qSOFA, quick sequential organ failure assessment score.

Normally distributed continuous data are displayed as mean (standard deviation) and compared using Welch’s t¬-test; non-normally distributed continuous data are displayed as median [interquartile range]; categorical data are displayed as count (percentage) and compared using Fisher’s exact test.

* Measured upon presentation to the emergency ward.

† Pathogens identified: influenza A (n = 4), Pseudomonas aeruginosa (n = 4), Streptococcus pneumoniae (n = 3), Moraxella catarrhalis (n = 2), Staphylococcus aureus (n = 2), Haemophilus influenzae (n = 1), rhinovirus (n = 1), respiratory syncytial virus (n = 1), and Aspergillus spp. (n = 1).

Co-infections in three patients: (1) Pseudomonas aeruginosa and Staphylococcus aureus, (2) Streptococcus pneumoniae and influenza A, and (3) Streptococcus pneumoniae and respiratory syncytial virus.

†† Defined as the modified Halm’s criteria (14): temperature ≤37,2°C, heart rate ≤100 bpm, systolic blood pressure ≤90 mmHg, respiratory rate ≤ 24 bpm, and oxygen saturation ≥90% for the entire day.

### Differential abundance of monocyte/dendritic cell and unconventional T cell metaclusters characterize the immune response in CAP

We identified 86 metaclusters (MCs) in PBMCs by 36-color spectral flow cytometry followed by unsupervised clustering using FlowSOM (12) (**Supplementary Figures 2, 3**). We classified these MCs into seven parent lineages: monocytes and dendritic cells (DCs), natural killer (NK) cells, B cells, CD4 T cells, CD8 T cells, unconventional T cells, and granulocytes (**Figures 1B–E** and **Supplementary Figure 4A**). The proportions of these lineages were similar between patients and controls, except for unconventional T cells, which were low in abundance in both groups, yet relatively less abundant in CAP patients (median for CAP, 1.8% [IQR, 1.2-2.6], median for control, 2.2% [IQR, 1.6-3.9]; *P* = 0.043; **Figure 1E** and **Supplementary Figure 4B**). We next compared the abundance of all 86 MCs between patients and controls. This untargeted analysis resulted in 19 (22.1%) significantly differentially abundant MCs (BH-adjusted *P <*0.05) between groups (**Figure 1F**). MCs within the monocyte and DC lineage featured prominently in this comparison (7/19 [36.8%] of total MCs significantly different), as did unconventional T cell MCs (5/19 [26.3%]). MCs 50 (neutrophils) and 54 (basophils) are likely low density granulocytes that commonly contaminate PBMC fractions of patients with inflammatory conditions (15). These cells comprised only a minor fraction of total cells and will not be discussed further. As alterations in immune phenotypes have been reported when comparing patients with COPD with healthy controls (16), we repeated the comparison between patients with CAP and control subjects after excluding patients (n = 15) and controls (n = 2) with COPD. The effect sizes in this comparison correlated almost perfectly to the effect sizes of the whole cohort (Pearson’s r = 0.97, P <0.0001), indicating that the higher prevalence of COPD in patients with CAP did not confound the comparison of circulating immune cell phenotypes between CAP and control subjects (**Supplementary Figure 5**). Thus, substantial differences emerged in the relative abundance of immune cell MCs between patients with CAP and controls, driven primarily by monocytes, DCs and unconventional T cells.

### Monocytes and DCs express markers of immune suppression

**Figure 2:**
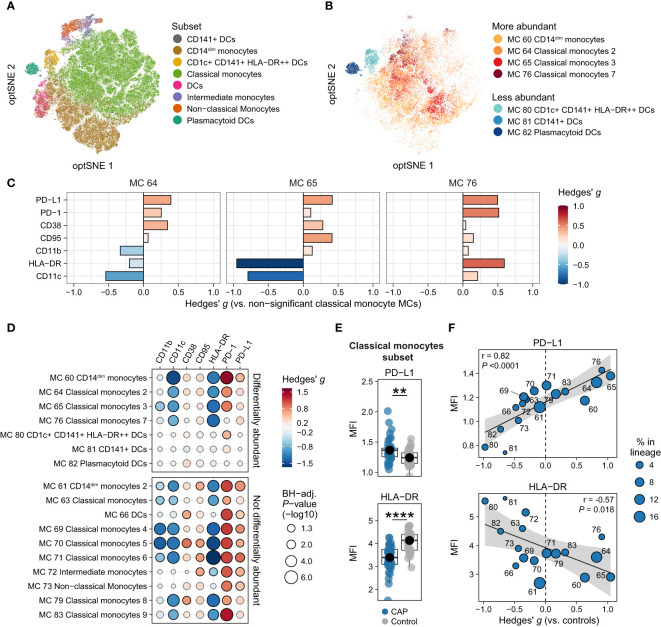
Immune activation and suppression markers on monocytes and dendritic cells (DCs). **(A)** Optimized t-distributed stochastic neighbor embedding (opt-SNE) plot representative of all monocytes and DCs, colored by cell subset (e.g. all nine classical monocyte metaclusters [MCs] have the same color). **(B)** opt-SNE plot with the same coordinates as shown in **(A)**, but only showing MCs significantly different between patients with CAP and controls in the monocyte/DC lineage. Shades of red indicate MCs significantly higher in CAP, shades of blue indicate MCs significantly higher in controls. **(C)** Expression of surface markers relevant for immune cell activation and suppression on individual cells in the three classical monocyte MCs increased in CAP (MCs 64, 65, and 76), as compared with all MCs not significantly different in **Figure 1F** (MCs 63, 69, 70, 71, 79, and 83). The difference in fluorescence intensity is expressed as a Hedges’ g effect size. The magnitude of this effect size is commonly interpreted as follows: ≥0.2 = small effect, ≥0.5 = moderate effect, ≥0.8 = large effect. **(D)** Bubble plot for the statistical comparisons between median fluorescence intensity (MFI) of the selected surface markers for each patient within each MC, i.e. using each individual subject’s MFI of their cells within that MC, and then comparing CAP and control. The size of the bubbles is proportional to the -log10-transformed BH-adjusted P value obtained using Wilcoxon’s rank-sum test (adjusted per surface marker). Non-significant comparisons are displayed as more transparent bubbles to facilitate interpretation of overall patterns. The color of the bubbles is proportional to the magnitude and direction of the differences (red is higher in CAP, blue is lower in CAP), expressed as the Hedges’ g effect size. The plot is stratified based on whether these MCs are differentially abundant in the volcano plot depicted in **Figure 1F**. **(E)** Boxplots showing the (arcsinh transformed) MFI of programmed death ligand 1 (PD-L1) and human leukocyte antigen – DR isotype (HLA-DR) for each subject within the classical monocyte subset (i.e. all nine classical monocyte MCs taken together). Each colored dot represents an individual subject, the box represents the lower and upper quartile, the middle line and black dot represent the median. **P <0.01, ****P <0.0001. **(F)** Scatterplots for the correlations between the expression of PD-L1 and HLA-DR and the relative abundance of monocyte/DC MCs. Y-axis shows the (arcsinh transformed) MFI; X-axis shows the Hedges’ g effect size (positive means higher in CAP versus controls, negative means lower in CAP versus controls). The correlation coefficient (Pearson’s r) and corresponding P-value are depicted in the plot. The size of the individual points in the scatterplots is proportional to the mean proportion of these cells within all monocytes and DCs of both CAP and control subjects. PD-1, programmed death 1.

We delineated 17 monocyte/DC MCs (**Figure 1D**), divided over 8 cell subsets (**Figure 2A**). The four monocyte/DC MCs that were significantly more abundant in patients with CAP compared with controls were three classical monocytes MCs and one CD14^dim^ monocyte MC, whereas all less abundant MCs within this lineage were DC MCs (**Figure 2B**; **Supplementary Figure 6A** for the relative abundance of all monocyte and DC MCs). We examined the functional profile of these MCs by assessing the expression of seven surface markers relevant for immune cell activation and suppression: CD11b, CD11c, CD38, CD95/Fas, human leukocyte antigen – DR isotype (HLA-DR), programmed death 1 (PD-1), and PD-ligand 1 (PD-L1; **Supplementary Figure 6B** for all markers). First, we compared the expression of these markers on the significantly more abundant classical monocyte MCs (MCs 64, 65 and 76) with the remaining non-significant classical monocyte MCs (MCs 63, 69, 70, 71, 79, and 83; **Figure 2C**). MCs 64, 65, and 76 all showed increased expression of PD-L1, and to a lesser extent PD-1 and CD38. Expression of HLA-DR was evidently decreased on MC 65, but increased on MC 76. CD11c was decreased on MCs 64 and 65, but slightly increased on MC 76. Concurrent expression of HLA-DR and CD11c on MC 76 may indicate that these cells are pro-inflammatory, whereas the concurrent downregulation of these molecules on MC 65 likely indicates an immunosuppressive phenotype. The differential expression patterns on the significantly more abundant classical monocyte MCs (**Figure 2C**) may reflect both immune suppression (e.g. reduced HLA-DR and increased PD-L1) and inflammatory activity (e.g. increased CD38 expression), which are both key features of the immunopathology in critically ill patients with sepsis (17).

We next contrasted expression patterns of these markers across all monocyte/DC MCs. For this, we compared the expression (as median fluorescence intensity [MFI] per subject) between CAP and control cells *within* each MC (**Figure 2D**) and each subset (**Figure 2E** and **Supplementary Figure 7**). These analyses revealed that cells from patients with CAP exhibited markedly decreased expression of HLA-DR – even in MC 76, which had an overall high expression of HLA-DR compared with other classical monocyte MCs (**Figure 2C**) – and CD11c, and increased expression of PD-1 and PD-L1. To a lesser extent, CD11b was reduced, and CD38 and CD95/Fas increased. We then explored whether the expression of these markers corresponds to the relative abundance of monocyte/DC MCs (**Supplementary Figure 8**). We found linear relationships between the overall expression of PD-L1 and HLA-DR (the MFI for all cells together) and the relative abundance of each MC (quantified as the Hedges’ *g* effect size compared with control); for example, the more abundant a monocyte/DC MC was in CAP, the higher the expression of PD-L1 (Pearson’s *r* = 0.82; *P <*0.0001), and the lower the expression of HLA-DR (*r* = -0.57; *P* = 0.018; **Figure 2F**). Taken together, these results indicate that, when compared with control subjects, monocytes and DCs from patients with CAP display variable signs of inflammation-induced activation, yet clear subset-wide signs of immune suppression (increased PD-L1 and PD-1, lower HLA-DR) and susceptibility to (Fas ligand-mediated) apoptosis (increased CD95/Fas).

### Universal upregulation of CCR7 and CXCR5 on monocytes and DCs

**Figure 3:**
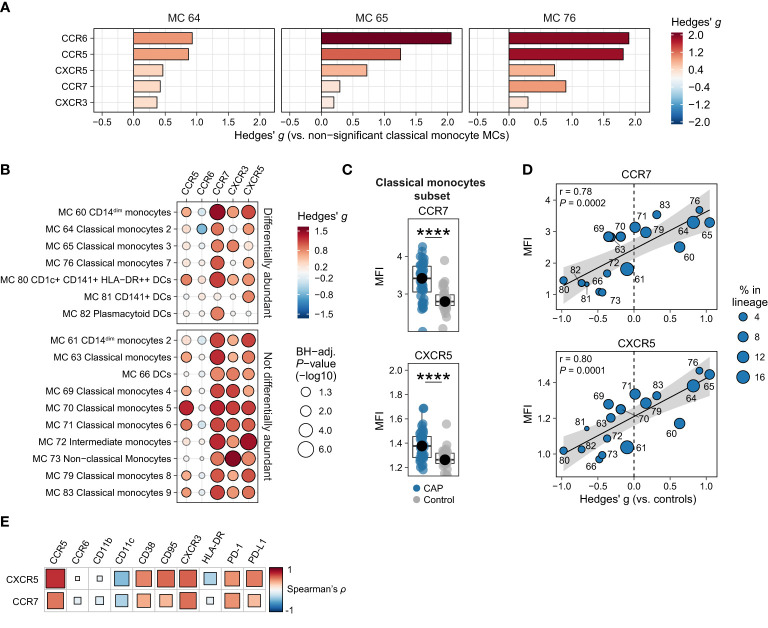
Chemokine receptors on monocytes and dendritic cells (DCs). **(A)** Expression of chemokine receptors on individual cells in the three classical monocyte MCs increased in CAP (MCs 64, 65, and 76), as compared with all MCs not significantly different in **Figure 1F** (MCs 63, 69, 70, 71, 79, and 83). The difference in fluorescence intensity is expressed as a Hedges’ g effect size. The magnitude of this effect size is commonly interpreted as follows: ≥0.2 = small effect, ≥0.5 = moderate effect, ≥0.8 = large effect. **(B)** Bubble plot for the statistical comparisons between median fluorescence intensity (MFI) of the selected surface markers for each patient within each metacluster (MC), i.e. using each individual subject’s MFI of their cells within that MC and then comparing CAP and control. The size of the bubbles is proportional to the -log10-transformed Benjamini-Hochberg- (BH)-adjusted P value obtained using Wilcoxon’s rank-sum test (adjusted per surface marker). Non-significant comparisons are displayed as transparent bubbles to facilitate interpretation of overall patterns. The color of the bubbles is proportional to the magnitude and direction of the differences (red is higher in CAP, blue is lower in CAP), expressed as the Hedges’ g effect size. The plot is stratified based on whether these MCs are differentially abundant in the volcano plot depicted in **Figure 1F**. **(C)** Boxplots showing the (arcsinh transformed) MFI of CCR7 and CXCR5 for each subject within the classical monocyte subset (i.e. all nine classical monocyte MCs taken together). Each colored dot represents an individual subject, the box represents the lower and upper quartile, the middle line and black dot represent the median. ****P <0.0001. **(D)** Scatterplots for the correlations between the expression of CCR7 and CXCR5 and the relative abundance of monocyte/DC MCs. Y-axis shows the (arcsinh transformed) MFI; X-axis shows the Hedges’ g effect size (positive means higher in CAP versus controls, negative means lower in CAP versus controls). The correlation coefficient (Pearson’s r) and corresponding P-value are depicted in the plot. The size of the individual points in the scatterplots is proportional to the mean proportion of these cells within all monocytes and DCs of both CAP and control subjects. **(E)** Plot for correlations between CCR7 and CXCR5 expression and the expression of other selected surface markers on all nine classical monocyte MCs. The size and color of the square are proportional to the correlation coefficient (Spearman’s *ρ*). Red represents a positive correlation, blue a negative correlation. PD-(L)1, programmed death (ligand) 1.

Considering their importance in immune cell migration, we next examined the expression of chemokines receptors. The three more abundant classical monocyte MCs all showed increased expression of all measured chemokine receptors (CCR5, CCR6, CCR7, CXCR3, and CXCR5), when compared with the classical monocyte MCs that were not differentially abundant (**Figure 3A**). However, when comparing the MFI for these chemokine receptors between CAP and controls for each subject within each MC (**Figure 3B**) and each subset (**Figure 3C** and **Supplementary Figure 9**), we found a near-universal upregulation of the chemokine receptors CXCR3, CCR7, and CXCR5 across most monocyte/DC MCs and cell subsets in patients with CAP. In addition, we found strong linear relationships between the relative abundance of monocyte/DC MCs and the overall expression of CCR7 (*r* = 0.78; *P* = 0.0002) and CXCR5 (*r* = 0.80; *P* = 0.0001; **Figure 3D**); such relationships were not present for the other chemokine receptors (**Supplementary Figure 8**). Expression of CCR7 and CXCR5 positively correlated with PD-1, PD-L1 and CD95/Fas, and negatively correlated with CD11c and to a lesser extent HLA-DR (**Figure 3E**), and thus appear related to the immunosuppressed and apoptosis-susceptible monocyte/DC phenotype described in **Figure 2**.

### Classical monocyte phenotypes are associated with systemic inflammation and clinical outcomes

**Figure 4:**
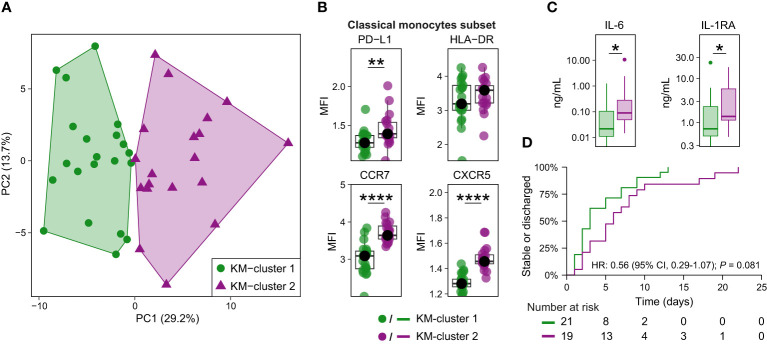
Classical monocyte surface marker expression delineates two clusters of patients with CAP. **(A)** Biplot for principal component (PC)1 and PC2 obtained by principal component analysis using the median expression for each patient of all surface markers indicative of immune cell activation, suppression, and migration on the nine classical monocyte clusters. K-means clustering defined the two clusters, referred to as KM-cluster 1 (n = 21) and KM-cluster 2 (n = 19). **(B)** Boxplots showing the (arcsinh transformed) median fluorescence intensity (MFI) per patient of depicted surface markers within the classical monocyte subset, stratified by the two KM-clusters. Each colored dot represents an individual subject, the box represents the lower and upper quartile, the middle line and black dot represent the median. **P <0.01, ****P <0.0001. **(C)** Boxplots for the plasma concentrations of IL-6 and IL-1RA patients with available plasma samples in KM-cluster 1 (n = 20) and KM-cluster 2 (n = 17). The box represents the lower and upper quartile, the middle line represent the median, the whiskers represent the distribution up to 1.5 times the interquartile range beyond the lower or upper quartile, outliers are depicted as individual dots. *P <0.05. **(D)** Kaplan-Meier curve for the time-to-event analysis of time to clinical stability or discharge. Clinical stability was defined using the modified Halm’s criteria (14): temperature ≤37,2°C, heart rate ≤100 bpm, systolic blood pressure ≤90 mmHg, respiratory rate ≤ 24 bpm, and oxygen saturation ≥90% for the entire day. The hazard ratio (HR) for KM-cluster 2 (compared with KM-cluster 1) from the Cox proportional hazards model is displayed in the graph. There were no competing risks for reaching clinical stability or discharge in this cohort (i.e. no patients were transferred or died prior to the event taking place). HLA-DR, human leukocyte antigen – DR isotype; IL(-RA), interleukin (receptor antagonist); PD-(L)1, programmed death (ligand) 1.

We next explored whether K-means clustering – an unsupervised machine learning algorithm – could use patterns of surface marker expression on classical monocyte MCs to distinguish subgroups of patients with CAP. The expression of CD11c, CD11b, CD38, CD95/Fas, PD-1, PD-L1, HLA-DR, CCR5, CCR6, CCR7, CXCR3, and CXCR5 on classical monocyte MCs separated patients in two distinct groups (KM-cluster 1, n = 21, and KM-cluster 2, n = 19; **Figure 4A** and **Supplementary Figure 10A**). Separation of the two KM-clusters was driven primarily by expression of CCR7 and CXCR5 (**Supplementary Figure 10B**). Consistently, when we compared the overall classical monocyte phenotype between the two KM-clusters, KM-cluster 2 was predominantly characterized by increased expression of chemokine receptors, except for CCR6 (**Figure 4B** and **Supplementary Figure 10C**). Remarkably, KM clustering using only CCR7 or CXCR5 resulted in almost the exact same cluster assignment for patients (**Supplementary Table 2**), emphasizing the key role of CCR7 and CXCR5 in separating these two groups of patients with CAP.

Patients in KM-cluster 2 had a longer time from start of symptoms to hospital admission (median, 4 vs 2 days; *P* = 0.03), and higher admission C-reactive protein (median, 235 vs 74 mg/L; *P* = 0.03; **Supplementary Table 3**). We next compared host response plasma biomarkers between these clusters of patients. While limited in statistical power due to sample size, the overall pattern suggested an exaggerated inflammatory state in KM-cluster 2 – consistent with the elevated C-reactive protein at hospital admission – with significantly higher IL-6 and IL-1RA levels (**Figure 4C** and **Supplementary Figure 10D**). Patients in KM-cluster 2 had an increase, albeit not statistically significant, in time to clinical stability or discharge (median, 6 vs 3 days; hazard ratio 0.56; 95% confidence interval 0.29-1.07; *P* = 0.08; **Figure 4D**). Together, this exploratory analysis provides preliminary evidence that classical monocyte phenotypes – in particular high expression of CCR7 and CXCR5 – are associated with the inflammatory status and disease course in patients with CAP.

### Other differentially abundant metaclusters in patients with CAP

**Figure 5:**
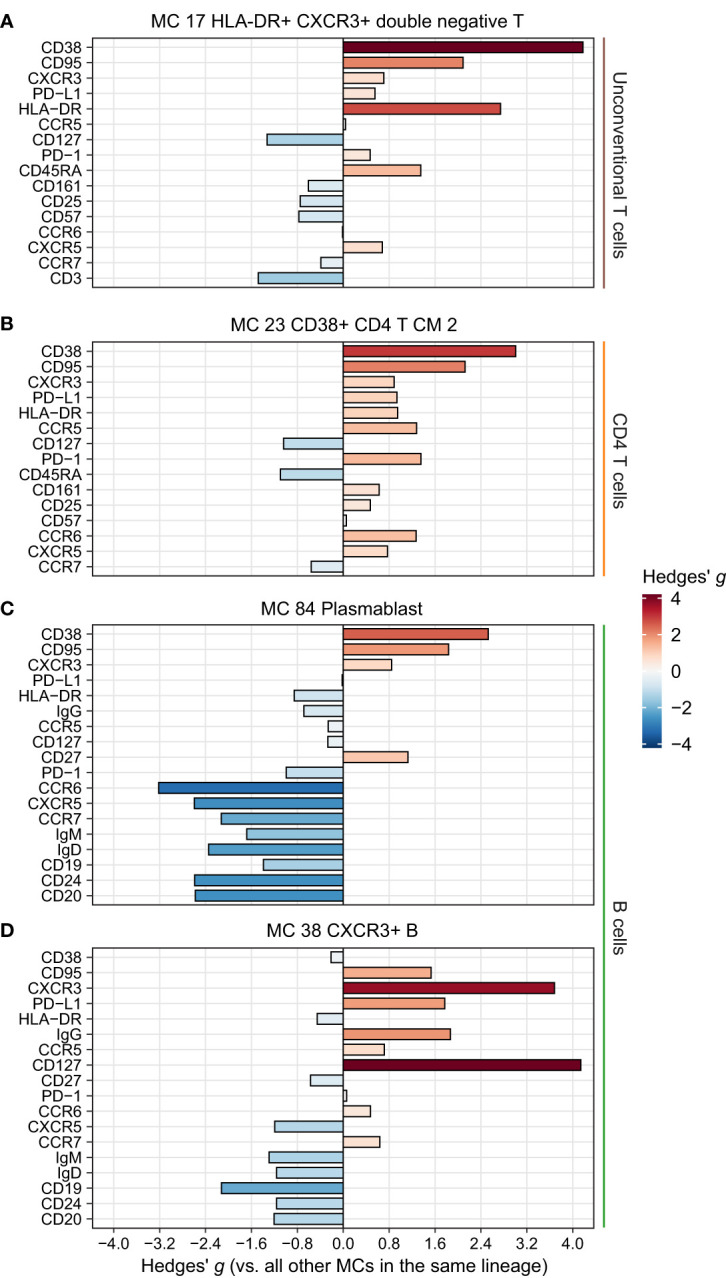
Phenotype of the remaining significantly more abundant metaclusters (MCs) in CAP. Expression of selected surface markers relevant for cell phenotype and function within the indicated lineages for MCs **(A)** 17, **(B)** 23, **(C)** 38, and **(D)** 86. The difference in fluorescence intensity is expressed as a Hedges’ g effect size obtained by comparing individual cells within the indicated MCs against all other cells within their respective lineages. HLA-DR, human leukocyte antigen – DR isotype; PD-(L)1, programmed death (ligand) 1.

Besides monocytes and DCs, other differentially abundant MCs – in the untargeted comparison of patients with CAP versus controls (**Figure 1F**) – included five unconventional T cell MCs: three less abundant TCRγδ T cell MCs (MCs 18, 24, 47), one less abundant double-positive T cell MC (MC 10), and one more abundant double-negative T cell MC (MC 17) with an activated phenotype (high HLA-DR, CD38 and CD95 expression; **Figure 5A**). Double-negative T cells are heterogeneous and can be either TCRαβ+ or TCRγδ+ and may be both immunosuppressive and proinflammatory (18, 19). While data on double-negative T cells in pneumonia are limited, increased circulating numbers have been reported to correlate with disease severity in pediatric pneumonia (20) – specifically the CD3^low^ subset, which may correspond to the relatively low CD3 expression in MC 17 – and also in COVID-19 (21).

For the remaining differentially abundant MCs (**Figure 1F**), we focused on those increased in patients with CAP. These included a CD4 central memory T cell cluster with an activated and possibly exhausted phenotype (high CD38, HLA-DR, PD-1, and CD95; **Figure 5B**); a plasmablast MC (MC 84, **Figure 5C**); and a B cell MC (MC 38) with high CXCR3 and high IgG, possibly reflecting an activated, class-switched pre-plasmablast/plasma-cell memory B cell (**Figure 5D**) (22). These results highlight that cells throughout the circulating immune system are altered in patients with CAP, and may point towards a hitherto underexplored role for unconventional T cells in CAP pathophysiology.

### Immune features largely overlap between patients with CAP and COVID-19

We next assessed to what degree peripheral immune features in patients hospitalized for CAP overlap with those in 35 patients hospitalized for COVID-19, with – similar to CAP – a low to moderate severity of disease (**Supplementary Table 4**). The proportions of lineages between COVID-19 and controls were similar (**Supplementary Figure 11A**). Differences in the relative abundance of MCs between COVID-19 patients and controls were largely in line with the COVID-19 peripheral immune signature reported in literature (23–31) (**Supplementary Figure 11B**). Specifically, for the innate immune system, this included an increase in classical monocytes (MCs 64 and 65) and neutrophils (MC 50, likely low density), with a concurrent decrease in non-classical and intermediate monocytes (MCs 72 and 73) and DCs (MCs 66 and 80); for the adaptive immune system this included an increase in plasmablasts (MC 86) and activated effector CD8 T cells (MC 33).

**Figure 6:**
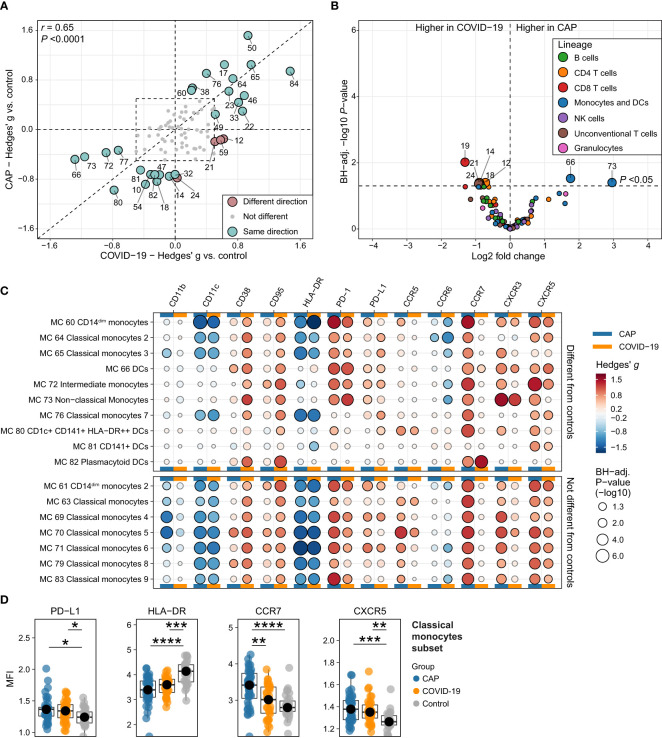
Circulating immune cell frequencies and phenotypes in CAP when compared with COVID-19. **(A)** Scatterplot displaying the difference in the abundance, expressed as a Hedges’ g effect size, for all 86 metaclusters (MCs) for CAP compared with controls (more towards the top on the Y-axis means higher in CAP) and for COVID-19 compared with controls (more to the right on the X-axis means higher in COVID-19). MCs with a biologically relevant difference with controls in one or both groups – defined as a Hedges’ g ≥0.5, indicated by the dashed line rectangle in the center – are highlighted as larger circles and labelled. The color indicates whether the metaclusters are in the same quadrant, i.e. whether the frequency of the MC shifts in the same direction when comparing CAP or COVID-19 with controls. The closer a circle is to the dashed diagonal line with a slope of 1, the closer the effect sizes for CAP or COVID-19 versus controls. The correlation between the effect sizes for all MCs is displayed as Pearson’s r in the top left corner. **(B)** Volcano plot for the comparison of all 86 MCs (as proportions of total number of cells per subject) between patients with CAP and patients with COVID-19. The X-axis depicts the difference in means of the log2-transformed proportion of each MC, the Y-axis depicts the -log10-transformed Benjamini-Hochberg- (BH)-adjusted P-value obtained using Welch’s t-test. Larger labelled points above the horizontal line represent significantly differentially abundant MCs. **(C)** Bubble plot for the statistical comparisons between MFI of the selected surface markers for each patient within each MC, i.e. using each individual subject’s MFI of their cells within that MC and then comparing CAP and control (in the columns marked by the horizontal blue bars) or COVID-19 and control (in the columns marked by the horizontal yellow bars). The size of the bubbles is proportional to the -log10-transformed BH-adjusted P value obtained using Wilcoxon’s rank-sum test (adjusted per surface marker). Non-significant comparisons are displayed as transparent bubbles to facilitate interpretation of overall patterns. The color of the bubbles is proportional to the magnitude and direction of the differences (red is higher in CAP versus control or COVID-19 versus control, blue is lower in CAP versus control or COVID-19 versus control), expressed as the Hedges’ g effect size. The bubbles for CAP are the same as displayed in **Figures 2E** and **Figures 3B**. The plot is stratified based on whether these MCs exhibit a biologically relevant difference with controls, as shown in **(A)**. **(D)** Boxplots showing the (arcsinh transformed) MFI of programmed death ligand 1 (PD-L1), human leukocyte antigen – DR isotype (HLA-DR), CCR7 and CXCR5 for each subject within the classical monocyte subset (i.e. all nine classical monocyte MCs taken together) for patients with CAP, patients with COVID-19, and controls. Statistical significance determined using a Kruskal-Wallis test, followed by a Bonferroni-adjusted pairwise Dunn’s test. Each colored dot represents an individual subject, the box represents the lower and upper quartile, the middle line and black dot represent the median. *P <0.05, **P <0.01, ***P <0.001, ****P <0.0001. PD-1, programmed death 1.

We assessed whether the immune features documented in patients with CAP were also present in patients with COVID-19. Interestingly, the majority of MCs with a biologically relevant difference compared with controls (defined as Hedges’ *g* ≥0.5 versus controls in CAP or COVID-19) changed in the same direction for CAP and COVID-19 (26/30 [86.7%]), indicating a large overlap in peripheral immune response (**Figure 6A**). Indeed, the proportional changes across all MCs strongly correlated between patients with CAP and patients with COVID-19 (Pearson’s *r* = 0.65; *P <*0.001). Directly comparing CAP and COVID-19 in an untargeted manner resulted in a modest number (8/86 [9.3%]) of differentially abundant MCs (with BH-adjusted *P*-values mostly very close to 0.05), including higher frequencies of several CD4, CD8 and TCRγδ T cell MCs (MCs 12, 14, 18, 19, 21, and 24) in COVID-19 and higher frequencies of DCs (MC 66) and non-classical monocytes (MC 73) in CAP (**Figure 6B**). Together, these data show that the proportional change of circulating immune cell frequencies is highly similar between patients with CAP and patients with COVID-19.

Finally, we investigated whether key findings in monocytes and DCs in CAP were also present in COVID-19. Comparing COVID-19 and controls at the level of MFI for each subject *within* each MC and each subset revealed expression patterns highly comparable to CAP, such as decreased HLA-DR and CD11c, and increased PD-L1, PD-1, CD95/Fas, CD38 and CXCR5 (**Figures 6C, D**). However, in clear contrast with CAP, classical monocytes of patients with COVID-19 did not express more CCR7 than controls (**Figures 6C, D**). In summary, these comparisons show that the immune response – at the level of circulating immune cell frequencies and monocyte/DC surface marker expression – is highly similar between CAP and COVID-19, with the notable exception of CCR7 expression on classical monocytes.

## Discussion

In this study we aimed to characterize the peripheral immune response in patients with CAP at single-cell protein levels using 36-color spectral flow cytometry. Compared with matched controls without signs of acute infection, the most profound alterations in patients with CAP were in the frequency and phenotype of monocyte and DC MCs. Specifically, we demonstrate both an increased frequency in distinct classical monocyte MCs, as well as phenotypic alterations across virtually all classical monocyte MCs reminiscent of immunosuppression (low HLA-DR, high PD-1 and PD-L1) and clear upregulation of chemokine receptors, most notably CCR7 and CXCR5. In an exploratory analysis, expression patterns on classical monocytes, driven by CCR7 and CXCR5, separated patients into two KM-clusters with diverging inflammatory status and disease course. The peripheral immune response in patients with COVID-19 was highly comparable to patients with CAP, yet lacked the increased expression of CCR7 on classical monocytes.

Immunosuppression is a key feature of the immunopathology of sepsis (32, 33). Reduced expression of HLA-DR (important for antigen presentation) and increased expression of PD-L1 (an inhibitory immune checkpoint protein) on monocytes are used as surrogates for an immunosuppressive state and, in septic shock, have been linked to reduced cytokine secretion upon stimulation, secondary infections, and mortality (34–37). In line with previous studies by our group (8, 38, 39), these indicators of immunosuppression were already present at hospital admission in this cohort of patients with CAP of only moderate disease severity. It remains to be established whether signs of immunosuppression in patients hospitalized with moderately severe CAP relate to clinical outcomes such as new infections (1, 4), an association that has been made in patients with septic shock (17, 33). Our understanding of the association between immunological profiles and clinical consequences of pneumonia would be enhanced by future longitudinal studies documenting immune and clinical readouts before, during, and after pneumonia, although such investigations are logistically and methodologically challenging.

We report a remarkable upregulation in the expression of CCR7 and CXCR5 across virtually all circulating classical monocyte MCs. Additionally, separation of KM-clusters 1 and 2 was driven by these chemokine receptors, and high expression of CCR7 and CXCR5 on classical monocytes in KM-cluster 2 was associated with systemic inflammation and a clear trend towards a longer time to clinical stability. Moreover, enhanced CCR7 expression on classical monocytes was restricted to CAP and not present in COVID-19. CCR7 is best known for its role in the migration of DCs and T cells to lymph nodes for antigen presentation, and CXCR5 for migration of B and T cells to lymphoid follicles (40, 41). In agreement with our results, exposure of human monocytes to toll-like receptor agonists, β-adrenergic agents and supernatants of activated platelets, increased CCR7 and CXCR5 expression (42, 43). Particularly monocyte CCR7 may impact the host response during bacterial infection. CCR7 is considered a marker for pro-inflammatory M1 macrophages (44), and binding of CCR7 on monocytes/macrophages by its ligands CCL19 or CCL21 may potentiate the secretion of pro-inflammatory mediators such as tumor necrosis factor and IL-8 (45, 46). Patients with sepsis showed increased CCR7 expression on circulating monocytes (47, 48), and a bioinformatics analysis of whole blood leukocyte RNA expression in datasets of patients with CAP-induced sepsis implicated *CCR7* as a gene driving sepsis development (49). In mice with *Pseudomonas* pneumonia CCR7 deficiency resulted in a stronger proinflammatory response and a more efficient clearance of bacteria from the lungs (50). Collectively, these results identify monocyte CCR7 as a potentially important player in the host response during pneumonia.

This study is the first to comprehensively characterize PBMC immunophenotypes at single cell resolution in patients with CAP. We used two matched control groups, including patients with COVID-19, which allowed identification of common and distinctive immune features. Limitations include that measurements were performed at a single time point and in a single compartment (i.e. blood), and the lack of functional testing of cell subsets/MCs. Furthermore, while our panel was extensive, it does not capture all rare cell subsets. Although absence of the confounding influence of dexamethasone treatment in our comparison of CAP with COVID-19 can be considered a strength, results obtained in the latter group may be less generalizable to patients currently hospitalized with COVID-19. The K-means clustering analysis was aimed at linking immune phenotypes to clinical characteristics, but should be considered exploratory and requires validation in a larger cohort.

We provide a comprehensive immunological map of PBMCs in patients with moderately severe CAP at single-cell protein level, revealing monocyte MCs with immunosuppressive features and enhanced chemokine receptor expression, and disclosing increased classical monocyte CCR7 expression as a feature unique to CAP when compared with COVID-19. Our results may offer an entry point toward developing novel immunomodulatory treatments of patients with CAP.

## Data availability statement

The raw data supporting the conclusions of this article will be made available by the authors, without undue reservation.

## Ethics statement

The studies involving humans were approved by the medical ethical committee of the Amsterdam University Medical Centers. The studies were conducted in accordance with the local legislation and institutional requirements. The participants provided their written informed consent to participate in this study.

## Author contributions

TR: Conceptualization, Data curation, Formal Analysis, Investigation, Methodology, Project administration, Visualization, Writing – original draft, Writing – review & editing. ARS: Conceptualization, Data curation, Formal Analysis, Investigation, Methodology, Project administration, Visualization, Writing – original draft, Writing – review & editing. JV: Conceptualization, Data curation, Formal Analysis, Methodology, Software, Visualization, Writing – review & editing. MB: Formal Analysis, Writing – review & editing. RD: Investigation, Writing – review & editing. DF: Investigation, Writing – review & editing. AP: Data curation, Formal Analysis, Investigation, Software, Writing – review & editing. WW: Conceptualization, Funding acquisition, Investigation, Project administration, Supervision, Writing – review & editing. AS: Conceptualization, Formal Analysis, Investigation, Supervision, Writing – review & editing. JG: Conceptualization, Data curation, Formal Analysis, Funding acquisition, Investigation, Methodology, Resources, Software, Supervision, Visualization, Writing – review & editing. TP: Conceptualization, Funding acquisition, Investigation, Methodology, Project administration, Resources, Supervision, Writing – original draft, Writing – review & editing.
