# Supplementary material for: High-dimensional phenotyping of the peripheral immune response in community-acquired pneumonia
Source: Front Immunol. 2023 Nov 24;14:1260283. doi: 10.3389/fimmu.2023.1260283 (PMC10704504; doi:10.3389/fimmu.2023.1260283)
Supplement: Supplementary file 1 [file DataSheet_1.docx]

# Supplemental material

# High-dimensional phenotyping of the peripheral immune response in community-acquired pneumonia

Tom D.Y. Reijnders*, Alex R. Schuurman*, Jan Verhoeff, Marlous van den Braber, Renée A. Douma, Daniël R. Faber, Alberta G.A. Paul, W. Joost Wiersinga, Anno Saris, Juan J. Garcia Vallejo^†^, Tom van der Poll^†^

*These authors share first authorship

†These authors share senior authorship

# Supplemental Methods

## Participating hospitals and inclusion criteria

Participating hospitals were Amsterdam University Medical Centers, location Academic Medical Center and location Vrije Universiteit; BovenIJ Hospital (all in Amsterdam, The Netherlands), and Flevo Hospital (Almere, The Netherlands). Patients aged ≥18 were eligible for inclusion if they were admitted to the hospital with a suspicion of a community-acquired, non-aspiration, respiratory tract infection; at least one respiratory symptom (new cough or sputum production, chest pain, dyspnea, tachypnea, abnormal lung examination, or respiratory failure); at least one systemic symptoms (fever or hypothermia, leukocytosis or leukopenia); and evidence for new or progressive chest imaging abnormalities consistent with infection. Diagnosis of COVID-19 was further based on a positive RT-PCR for SARS-CoV-2, except in two patients in whom the diagnosis was made based on a clinical presentation compatible with COVID-19 and a CORADS score >4 (1). Patients were excluded if they received chemotherapy within the last six months, used high-dose and/or potent immunosuppressive drugs (e.g. for solid organ transplantation), or had an active hematological malignancy. Age- and sex-matched control subjects without signs of acute infection were recruited from the outpatient clinics. As our cases of patients with community-acquired pneumonia (CAP) and COVID-19 were highly similar in terms of disease severity scores, we did not further match them based on other clinical characteristics; differences between baseline characteristics were subtle and representative of the baseline disease presentations of CAP and COVID-19 known from previous literature (Table 1 and Supplemental Table 4) (2,3). This study did not interfere with routine clinical diagnostics, and all pathogens identified in patients with CAP were based on standard of care clinical microbiology results reported in patients’ electronic health records (e.g. blood cultures, sputum cultures, urinary antigen tests for *Streptococcus pneumoniae*, and polymerase chain reaction tests for [viral] pathogens such as influenza A). More detailed information on study procedures in the ELDER-BIOME study can be found in prior publications (4–6).

## Spectral flow cytometry staining and acquisition

We thawed and stained cryopreserved peripheral blood mononuclear cells (PBMCs) for spectral flow cytometry in five batches and split acquisition across three days. Each batch included a technical replicate to function as a reference control. Supplemental Table 1 shows antibodies used. Cryopreserved PBMCs were thawed and stained as previously described (7). In short, cells were thawed at 37 °C and washed with pre-warmed RPMI + 2mM L-GLUT and 10% fetal calf serum. After staining with LIVE/DEAD Fixable Blue Dead Cell stain kit (ThermoFisher Scientific, Waltham, Massachusetts), cells were washed and supernatant was aspirated. Brilliant Stain Buffer (Becton Dickinson, Franklin Lakes, New Jersey) and True-Stain Monocyte Blocker (Biolegend, San Diego, California) were added while the antibody cocktails were prepared. Cell suspensions were kept at 4 °C and protected from light. In sequence, anti-TCRγδ, anti-CCR7, and a combination of anti-CCR6, anti-CCR5, anti-CXCR5, and anti-CXCR3 were added to the cell suspensions in 10 min intervals. Lastly, the remaining antibodies were added to the cell suspensions. After washes, cells were fixed with 1% PFA (ThermoFisher Scientific, Waltham, Massachusetts) on ice. After the staining procedure, cells were kept at 4 °C for one-two days until acquisition on a five-laser Aurora spectral flow cytometer (Cytek Biosciences, Fremont, California).

## Data processing and analysis

Preprocessing of data included spectral unmixing, autofluorescence extraction, and spill-over correction through SpectroFlow software (Cytek Biosciences, Fremont, California). Data were uploaded to OMIQ (Dotmatics, Boston, Massachusetts) and an arcsinh transformation was applied. Co-factors for arcsinh were determined visually on a per marker basis. Further downstream analysis and reporting of data used the transformed data. Data were checked for consistency over time and non-conforming sections were gated out using flowCut (ref flowcut). Viable CD45+ single cells were selected by manual gating (a representative gating strategy is shown in Supplemental Figure 1). We sampled up to 400,000 live CD45+ cells per subject for analysis, the median number cells analyzed per sample was 391,521 (interquartile range [IQR], 239,247 – 400,000). To enable comparison of the three experimental groups (CAP, COVID-19, and controls), all samples were clustered together and underwent subsequent phenotyping.

Data were transferred to R. Batch effects were investigated and corrected for using the 5 reference control samples. A modified version of CytoNorm was used.(8) In short, reference controls were broadly clustered into 6 lineages: B cells, natural killer (NK) cells, CD4 T cells, CD8 T cells, monocytes, and remaining myeloid + CD45^dim^ events. Per lineage marker distributions were investigated. Positive peaks were well aligned, e.g. CD4 signal on CD4^+^ T cells or CD45RA on B cells. However several markers experienced batch effects in lineages where there was low expression; CD14 on T cell lineages, or CD20 in monocytes. For these poorly performing distributions the quantile normalization of CytoNorm was used.

Data were clustered using FlowSOM (9) (version 1.18) within R (version 3.6), into 600 SOM clusters. The optimal number of metaclusters (MCs) was determined through the use of INFLECT (10), a software package that checks the distributions of markers within MCs, for every possible number of MCs. Checks consisted of the dip-test, a statistical test for unimodality and an interquartile range test. The percentage of distributions that pass these checks was plotted (Supplemental Figure 2A) versus the given number of MCs, after which the inflection point 86 MCs – the number of MCs with the optimal trade-off between clustering performance and statistical power – was taken. Phenotypes of MCs were manually determined through investigation of median marker expression in combination with concordance with Uniform manifold approximation and projection (UMAP) plots. MCs were phenotyped by comparison to manual gating strategies described in the Optimized Multicolor Immunofluorescence Panel (OMIP) 069 (7).

## Statistical analysis

Data are reported as mean (standard deviation [SD]) for normally distributed continuous variables, as median (IQR) for non-normally distributed continuous variables, and as count (percentage) for categorical data. To assess differences in MC proportions in an untargeted manner (i.e. the volcano plot), we performed Welch’s t­-test on log2-transformed MC proportions (of total cells) and applied Benjamini-Hochberg- (BH-)adjustment to the resulting P-values. Prior to log2-transformation, MCs with a proportion of zero percent were imputed to half the lowest proportion measured for that MC. To compare the fluorescence intensity of surface markers between patient groups, we calculated the median fluorescence intensity for all cells of each patient within an MC and subsequently compared these medians using Wilcoxon’s rank-sum test (in case of two groups), or a Kruskal-Wallis test followed by a Bonferroni-adjusted Dunn’s post-hoc test (in case of three groups). To compare the fluorescence intensity of individual MCs with other MCs, we calculated the Hedges’ *g* effect size for each marker using all individual cells within that cluster to all individual cells in the comparator MCs. Plasma biomarker samples were not available in 3/40 (7.5%) of patients with CAP, these data were considered missing completely at random and not imputed.

The UMAP for Figure 1 was generated using a random subset of 1 million cells, with n_neighbours = 30 and min_dist = 0.25, and the standard settings of the UMAP function within the *uwot* R package. Optimized t-SNE (opt-SNE) plots(11) were generated using OMIQ, and included all monocytes and dendritic cells randomly selected for the Figure 1 UMAP. We performed principal component analysis using *mixOmics* package with nonlinear iterative partial least squares (NIPALS) algorithm (standard setting) for missing data. K-means clustering of patients was performed with a fixed seed, on NIPALS-imputed, scaled and centered expression data of selected surface markers on all classical monocyte MCs. The optimal number of K-means (KM-)clusters was determined based on a silhouette plot generated with the fviz_nbclust function within the *factoextra* package. The differences in the rate of reaching clinical stability or discharge (i.e. the hazard ratio for KM-cluster 2 compared relative to KM-cluster 1) was estimated using a Cox proportional hazards model. A model taking into account the potential competing events of mortality or transfer to a different hospital was not necessary, as these potential competing events did not occur in the cohort for this study.

## R packages

Analyses were performed in R (version 4.1.2). We used the following packages for statistical analyses: *rstatix, uwot, factoextra, NbClust, mixOmics, cluster*.

# References

1. Prokop M, van Everdingen W, van Rees Vellinga T, van Ufford HQ, Stöger L, Beenen L, Geurts B, Gietema H, Krdzalic J, Schaefer-Prokop C, et al. CO-RADS: A Categorical CT Assessment Scheme for Patients Suspected of Having COVID-19-Definition and Evaluation. *Radiology* (2020) 296:E97–E104. doi: 10.1148/RADIOL.2020201473

2. Torres A, Cilloniz C, Niederman MSM, Al. E, Menéndez R, Chalmers JD, Wunderink RG, van der Poll T. Pneumonia. (2021) 7:1–28. doi: 10.1038/s41572-021-00259-0

3. Wiersinga W, Rhodes A, Cheng A, Peacock S, Prescott HC. Pathophysiology, transmission, diagnosis, and treatment of coronavirus disease 2019 (COVID-19): a review. *JAMA - J Am Med Assoc* (2020) 324:782–793.

4. Schuurman AR, Reijnders TDY, Saris A, Moral IR, Schinkel M, de Brabander J, van Linge C, Vermeulen L, Scicluna BP, Wiersinga WJ, et al. Integrated single-cell analysis unveils diverging immune features of covid-19, influenza, and other community-acquired pneumonia. *Elife* (2021) 10: doi: 10.7554/eLife.69661

5. Brands X, Haak BW, Klarenbeek AM, Otto NA, Faber DR, Lutter R, Scicluna BP, Wiersinga WJ, van der Poll T. Concurrent Immune Suppression and Hyperinflammation in Patients With Community-Acquired Pneumonia. *Front Immunol* (2020) 11: doi: 10.3389/FIMMU.2020.00796

6. Schuurman AR, Reijnders TDY, van Engelen TSR, Leopold V, de Brabander J, van Linge C, Schinkel M, Pereverzeva L, Haak BW, Brands X, et al. The host response in different aetiologies of community-acquired pneumonia. *EBioMedicine* (2022) 81:104082. doi: 10.1016/J.EBIOM.2022.104082

7. Park LM, Lannigan J, Jaimes MC. OMIP-069: Forty-Color Full Spectrum Flow Cytometry Panel for Deep Immunophenotyping of Major Cell Subsets in Human Peripheral Blood. *Cytometry Part A* (2020) 97:1044–1051. doi: 10.1002/CYTO.A.24213

8. van Gassen S, Gaudilliere B, Angst MS, Saeys Y, Aghaeepour N. CytoNorm: A Normalization Algorithm for Cytometry Data. *Cytometry Part A* (2020) 97:268–278. doi: 10.1002/CYTO.A.23904

9. van Gassen S, Callebaut B, van Helden MJ, Lambrecht BN, Demeester P, Dhaene T, Saeys Y. FlowSOM: Using self-organizing maps for visualization and interpretation of cytometry data. *Cytometry A* (2015) 87:636–645. doi: 10.1002/CYTO.A.22625

10. Verhoeff J, Abeln S, Garcia-Vallejo JJ. INFLECT: an R-package for cytometry cluster evaluation using marker modality. *BMC Bioinformatics* (2022) 23:1–15. doi: 10.1186/S12859-022-05018-W/FIGURES/5

11. Belkina AC, Ciccolella CO, Anno R, Halpert R, Spidlen J, Snyder-Cappione JE. Automated optimized parameters for T-distributed stochastic neighbor embedding improve visualization and analysis of large datasets. *Nat Commun* (2019) 10:1–12. doi: 10.1038/s41467-019-13055-y

12. Halm EA, Fine MJ, Marrie TJ, Coley CM, Kapoor WN, Obrosky DS, Singer DE. Time to Clinical Stability in Patients Hospitalized With Community-Acquired Pneumonia: Implications for Practice Guidelines. *JAMA* (1998) 279:1452–1457. doi: 10.1001/JAMA.279.18.1452

# Supplemental Tables

### Supplemental Table 1. Overview of antibodies for spectral flow cytometry

| Item | Fluorochome | Catalog # | Vendor | Unit Size | uL/test |
| --- | --- | --- | --- | --- | --- |
| Brilliant Stain Buffer Plus | N/A | 566385 | BD | 1000 tests | 10 ul |
| LIVE/DEAD™ Fixable Blue Dead Cell Stain Kit, for UV excitation | N/A | L34962 | Thermo | 400 assays | 5ul dil 1/40 |
| True-stain Monocyte Block |  |  | Biolegend |  | 5 ul |
| UV LASER |  |  |  |  |  |
| BUV395 Mouse Anti-Human CD45RA | BUV395 | 740315 | BD | 50 µg | 1.2 |
| BUV496 Mouse Anti-Human CD16 | BUV496 | 612944 | BD | 100 tests | 0.6 |
| BUV563 Mouse Anti-Human CD195 (CCR5) | BUV563 | 741401 | BD | 50ug | 2.5 |
| BUV661 Mouse Anti-Human CD11c | BUV661 | 612967 | BD | 100 tests | 2.5 |
| BUV737 Mouse Anti-Human CD56 | BUV737 | 612766 | BD | 100 tests | 1.2 |
| BD Horizon™ BUV805 Mouse Anti-Human CD8 | BUV805 | BD | 100 tests | 500 | 1.2 |
| VIOLET LASER |  |  |  |  |  |
| Brilliant Violet 421™ anti-human CD197 (CCR7) Antibody | BV421 | 353208 | Biolegend | 100 tests | 5 |
| CD123 Monoclonal Antibody (6H6), Super Bright 436, eBioscience™ | Super Bright 436 | 62-1239-42 | Thermo | 100 tests | 2.5 |
| CD161 Monoclonal Antibody (HP-3G10), eFluor 450, eBioscience™ | eFluor450 | 48-1619-42 | Thermo | 100 tests | 5 |
| BV480 Mouse Anti-Human IgD | BV480 | BD | 100 tests | 500 | 0.6 |
| Brilliant Violet 510™ anti-human CD3 Antibody | BV510 | 317332 | Biolegend | 100 tests | 5 |
| Brilliant Violet 570™ anti-human IgM Antibody | BV570 | 314517 | Biolegend | 25 tests | 2.5 |
| CD20 Monoclonal Antibody (HI47), Pacific Orange | Pacific Orange | Thermo | 0.5ml | 500 | 2.5 |
| BD Horizon™ BV605 Mouse Anti-Human IgG | BV605 | 563246 | BD | 50 tests | 5 |
| Brilliant Violet 650™ anti-human CD28 Antibody (clone CD28.2) | BV650 | 302946 | Biolegend | 100 tests | 2.5 |
| Brilliant Violet 711™ anti-human CD196 (CCR6) Antibody | BV711 | 353436 | Biolegend | 100 tests | 1.2 |
| BV750 Rat Anti-Human CXCR5 (CD185) | BV750 | 747111 | BD | 50 µg | 1.2 |
| Brilliant Violet 785™ anti-human CD279 (PD-1) Antibody | BV785 | 329930 | Biolegend | 100 tests | 5 |
| BLUE LASER |  |  |  |  |  |
| BD Horizon™ BB515 Mouse Anti-Human CD141 | BB515 | 566017 | BD | 25 tests | 2.5 |
| FITC anti-human CD57 Antibody | FITC | 359604 | Biolegend | 100 tests | 1.2 |
| Spark Blue™ 550 anti-human CD14 Antibody | SparkBlue550 | 367148 | Biolegend | 100 tests | 2.5 |
| PerCP anti-human CD45 Antibody | PerCP | 368506 | Biolegend | 100 tests | 2.5 |
| PerCP/Cyanine5.5 anti-human CD11b Antibody | PerCPCy5.5 | 301328 | Thermo | 100 tests | 5 |
| TCR gamma/delta Monoclonal Antibody (B1.1), PerCP-eFluor 710, eBioscience™ | PerCP-eF710 | 46-9959-42 |  | 100 tests | 1.2 |
| YELLOW/GREEN LASER |  |  |  |  |  |
| PE anti-human CD274 (B7-H1, PD-L1) Antibody | PE | 329706 |  | 100 ug | 1/200 titrated; 0.5 ul |
| CD4 CF568 | CF568 |  | Biolegend | 100 tests | 1.2 |
| PE/Dazzle™ 594 anti-human CD24 Antibody | PEDz594 | Biolegend | 100 tests | 500 | 5 |
| PE/Cy5 anti-human CD95 (Fas) Antibody | PECy5 | 305610 | Thermo | 100 tests | 1.25 |
| PE-Alexa Fluor 700 anti-human CD25 Antibody | PE-AF 700 | MHCD2524 | Biolegend | 100 tests | 5 |
| PE/Cy7 anti-human CD183 (CXCR3) Antibody | PECy7 | 353720 | Biolegend | 100 tests | 2.5 |
| RED LASER |  |  |  |  |  |
| APC anti-human CD27 Antibody | APC | 356410 | Biolegend | 100 tests | 2.5 |
| Alexa Fluor® 647 anti-human CD1c Antibody | AF647 | 331510 | Biolegend | 100 tests | 5 |
| Spark NIR™ 685 anti-human CD19 Antibody | SparkNIR685 | 302270 | Biolegend | 100 tests | 1.2 |
| APC/Fire™ 750 anti-human HLA-DR Antibody | APCF750 | 307658 | BD | 100 tests | 1.2 |
| APC-R700 Mouse Anti-Human CD127 | APC-R700 | 565185 | Biolegend | 50 tests | 6 |
| CD38 APC-Fire810 | APC-Fire810 | Custom | Biolegend |  | 1 |

### Supplemental Table 2. K-means cluster assignments per patient

| **Patient ID** | **Original KM-clusters** | **CCR7 only**  **KM-clusters** | **CXCR5 only**  **KM-clusters** |
| --- | --- | --- | --- |
| 1096 | 1 | 1 | 1 |
| 1097 | 2 | 2 | 2 |
| 1100 | 2 | 2 | 2 |
| 1101 | 2 | 2 | 2 |
| 1103 | 1 | 1 | 1 |
| 1106 | 1 | 2 | 1 |
| 1107 | 2 | 2 | 2 |
| 1109 | 2 | 2 | 1 |
| 1110 | 1 | 1 | 1 |
| 1112 | 1 | 1 | 1 |
| 1114 | 1 | 1 | 1 |
| 1115 | 1 | 1 | 1 |
| 1116 | 2 | 2 | 2 |
| 1118 | 2 | 2 | 2 |
| 1119 | 2 | 2 | 2 |
| 1120 | 1 | 1 | 1 |
| 1125 | 1 | 1 | 1 |
| 3033 | 2 | 2 | 2 |
| 3034 | 1 | 1 | 1 |
| 3035 | 1 | 1 | 1 |
| 3036 | 2 | 2 | 2 |
| 3037 | 1 | 1 | 1 |
| 3038 | 2 | 2 | 2 |
| 3039 | 1 | 1 | 1 |
| 3041 | 2 | 2 | 2 |
| 3043 | 1 | 1 | 1 |
| 3044 | 1 | 2 | 1 |
| 3045 | 1 | 1 | 1 |
| 3046 | 1 | 1 | 1 |
| 3047 | 1 | 1 | 1 |
| 3049 | 2 | 2 | 2 |
| 3050 | 1 | 1 | 1 |
| 3051 | 2 | 2 | 2 |
| 3053 | 1 | 2 | 2 |
| 3054 | 2 | 2 | 2 |
| 3055 | 2 | 2 | 2 |
| 3056 | 2 | 2 | 1 |
| 7001 | 2 | 2 | 2 |
| 7002 | 2 | 2 | 2 |
| 7005 | 1 | 1 | 1 |
| Patients that switch cluster from their original assignment are highlighted in yellow. | | | |

### Supplemental Table 3. Baseline characteristics and outcomes for KM-clusters 1 and 2

|  | **KM-cluster 1**  **(n = 21)** | **KM-cluster 2**  **(n = 19)** | ***P*-value** |
| --- | --- | --- | --- |
| **DEMOGRAPHICS** |  |  |  |
| Age, years | 69.8 (14.9) | 67.7 (16.3) | 0.68 |
| Sex, male | 15 (71.4) | 9 (47.4) | 0.20 |
| **COMORBIDITIES** |  |  |  |
| Chronic obstructive pulmonary disease | 7 (33.3) | 8 (42.1) | 0.75 |
| Asthma | 5 (23.8) | 1 (5.3) | 0.19 |
| Hypertension | 8 (38.1) | 8 (42.1) | >0.99 |
| Diabetes mellitus, type 2 | 1 (4.8) | 3 (15.8) | 0.33 |
| Chronic kidney disease | 2 (9.5) | 1 (5.3) | >0.99 |
| **LABORATORY TESTS*** |  |  |  |
| Platelets, x10^9^ cells/L | 263.5 (135.5) | 284.8 (102.9) | 0.58 |
| Leukocytes, x10^9^ cells/L | 13.6 [11.0, 19.5] | 11.1 [8.7, 15.6] | 0.32 |
| Neutrophils, x10^9^ cells/L | 11.9 [9.0, 16.6] | 9.0 [6.6, 13.2] | 0.33 |
| Lymphocytes, x10^9^ cells/L | 0.8 [0.6, 1.2] | 0.7 [0.6, 1.4] | 0.95 |
| Neutrophil-to-lymphocyte ratio | 10.7 [8.4, 15.3] | 8.1 [6.2, 16.3] | 0.31 |
| C-reactive protein, mg/L | 74.0 [55.5, 261.0] | 235.0 [143.7, 282.0] | 0.025 |
| **VITAL SIGNS AND DISEASE SEVERITY*** |  |  |  |
| Mean arterial pressure, mmHg | 98.1 (13.5) | 92.1 (14.6) | 0.19 |
| Respiratory rate, bpm | 21 [19, 30] | 22 [18, 28] | >0.99 |
| Temperature, ℃ | 37.6 (1.5) | 38.1 (1.0) | 0.28 |
| Modified Early Warning Score | 3 [2, 5] | 3 [2, 4] | 0.54 |
| Pneumonia Severity Index | 4 [3, 4] | 3 [2, 4] | 0.08 |
| CURB-65 | 2 [1, 2] | 1 [0, 1] | 0.05 |
| qSOFA | 1 [0, 1] | 1 [0, 1] | 0.74 |
| **CLINICAL COURSE AND OUTCOMES** |  |  |  |
| Symptoms to admission, days | 2 [2, 3] | 4 [2, 6] | 0.025 |
| ICU stay (at any point during admission) | 2 (9.5) | 1 (5.3) | >0.99 |
| Hospital length of stay, days | 3 [2, 8] | 5 [3, 10] | 0.21 |
| Time to clinical stability^†^ or discharge, days | 3 [2, 7] | 6 [3, 8] | 0.07 |
| 28-day mortality | 0 (0) | 0 (0) |  |
| bpm = breaths/beats per minute; CURB-65 = confusion, blood urea nitrogen, respiratory rate, blood pressure, age 65 or older; KM = K-means; qSOFA = quick sequential organ failure assessment score.  Normally distributed continuous data are displayed as mean (standard deviation) and compared using Welch’s *t­*-test; non-normally distributed continuous data are displayed as median [interquartile range]; categorical data are displayed as count (percentage) and compared using Fisher’s exact test.  * Measured upon presentation to the emergency ward  ^†^ Defined as the modified Halm's criteria (12): temperature ≤37·2 °C, heart rate ≤100 bpm, systolic blood pressure ≤90 mmHg, respiratory rate ≤ 24 bpm, and oxygen saturation ≥90% for the entire day | | | |

### Supplemental Table 4. Baseline characteristics and outcomes for patients hospitalized for COVID-19* and control subjects

|  | **COVID-19**  **(n = 35)** | **Controls**  **(n = 31)** | ***P*-value** |
| --- | --- | --- | --- |
| **DEMOGRAPHICS** |  |  |  |
| Age, years | 60.3 (11.5) | 64.3 (15.6) | 0.24 |
| Sex, male | 17 (48.6) | 20 (64.5) | 0.22 |
| **COMORBIDITIES** |  |  |  |
| Chronic obstructive pulmonary disease | 2 (5.7) | 2 (6.5) | >0.99 |
| Asthma | 3 (8.6) | 1 (3.2) | 0.62 |
| Hypertension | 13 (37.1) | 15 (48.4) | 0.46 |
| Diabetes mellitus, type 2 | 9 (25.7) | 7 (22.6) | >0.99 |
| Chronic kidney disease | 0 (0.0) | 3 (9.7) | 0.10 |
| **LABORATORY TESTS*** |  |  |  |
| Platelets, x10^9^ cells/L | 260.7 (108.4) |  |  |
| Leukocytes, x10^9^ cells/L | 7.1 [5.7, 9.0] |  |  |
| Neutrophils, x10^9^ cells/L | 5.1 [3.9, 6.9] |  |  |
| Lymphocytes, x10^9^ cells/L | 1.02 [0.70, 1.46] |  |  |
| Neutrophil-to-lymphocyte ratio | 5.3 [2.8, 10.5] |  |  |
| C-reactive protein, mg/L | 100.0 [50.4, 146.2] |  |  |
| **VITAL SIGNS AND DISEASE SEVERITY**^†^ |  |  |  |
| Mean arterial pressure, mmHg | 95.5 (15.4) |  |  |
| Respiratory rate, bpm | 24 [21, 29] |  |  |
| Temperature, ℃ | 37.6 (1.2) |  |  |
| Modified Early Warning Score | 3 [3, 4] |  |  |
| Pneumonia Severity Index | 3 [2, 3] |  |  |
| CURB-65 | 1 [0, 2] |  |  |
| qSOFA | 1 [0, 1] |  |  |
| **CLINICAL COURSE AND OUTCOMES** |  |  |  |
| Symptoms to admission, days | 8 [6, 10] |  |  |
| ICU stay (at any point during admission) | 6 (17.1) |  |  |
| Hospital length of stay, days | 4 [3, 8] |  |  |
| Time to clinical stability^††^ or discharge, days | 4 [3, 7] |  |  |
| 28-day mortality | 4 (11.4) |  |  |
| COVID-19 = coronavirus disease 2019; bpm = breaths/beats per minute; CURB-65 = confusion, blood urea nitrogen, respiratory rate, blood pressure, age 65 or older; qSOFA = quick sequential organ failure assessment score.  Normally distributed continuous data are displayed as mean (standard deviation) and compared using Welch’s *t­*-test; non-normally distributed continuous data are displayed as median [interquartile range]; categorical data are displayed as count (percentage) and compared using Fisher’s exact test.  * Patients with COVID-19 were included between April and May 2020 (prior to the introduction of the Alpha variant, the availability of vaccines, and dexamethasone becoming standard of care)  ^†^Measured upon presentation to the emergency ward  ^††^ Defined as the modified Halm's criteria (12): temperature ≤37·2 °C, heart rate ≤100 bpm, systolic blood pressure ≤90 mmHg, respiratory rate ≤ 24 bpm, and oxygen saturation ≥90% for the entire day | | | |

# Supplemental Figures


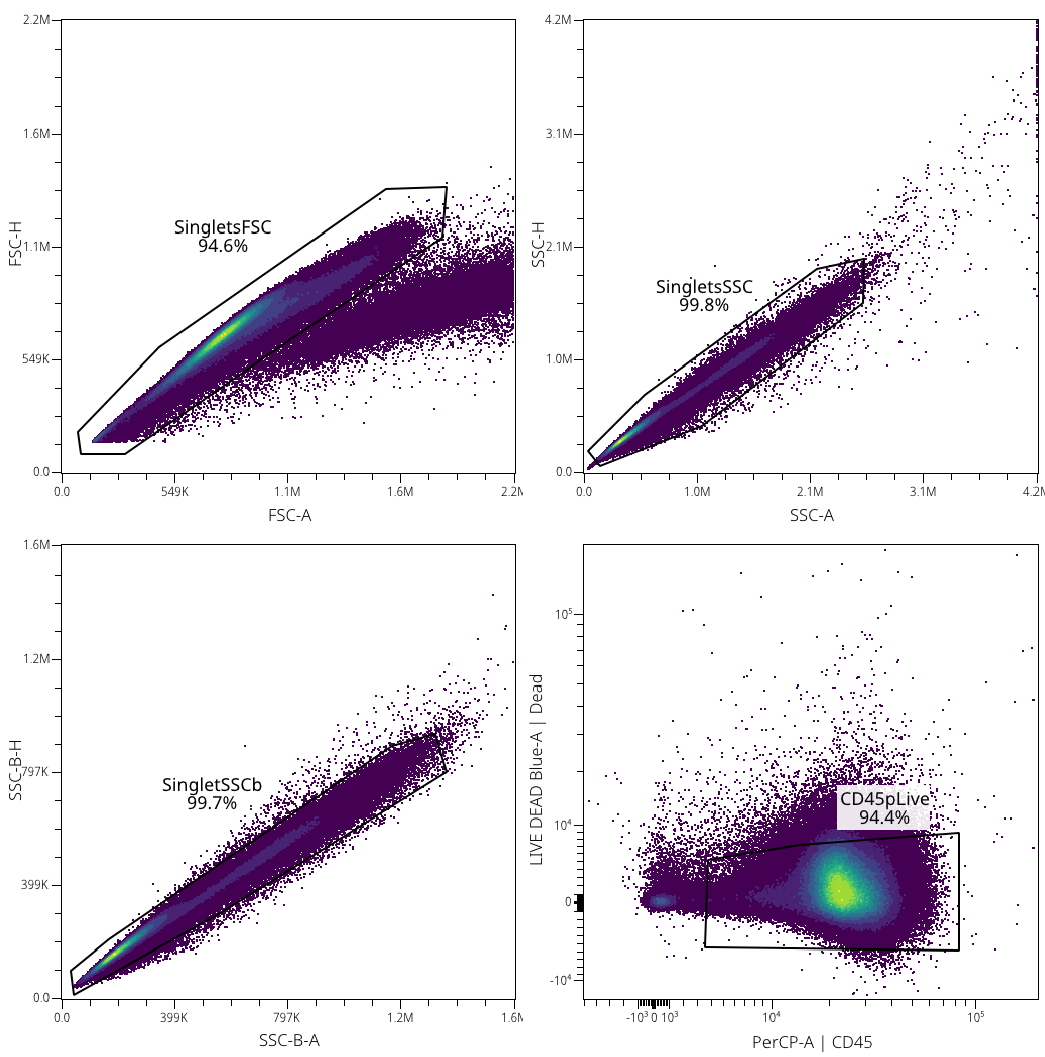


### Supplemental Figure 1

Representative gating strategy for selecting CD45+ live single cells per subject for subsequent analyses. FSC = forward scatter; SSC = side scatter.


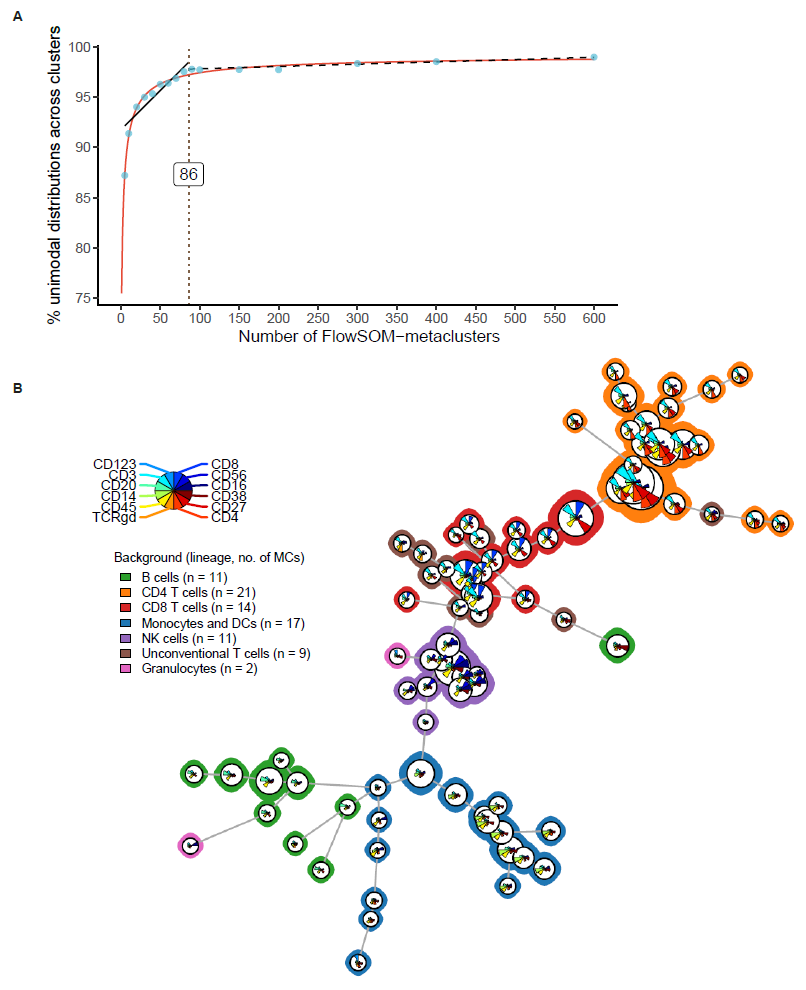


### Supplemental Figure 2

*(A*) Curve generated using the software package INFLECT (10) to determine the optimal number of metaclusters, as described in the Supplemental Methods. (*B*) FlowSOM tree depicting the 86 MCs obtained using cells from all subjects and with all available surface markers. Each MC is depicted as a circle with the following characteristics: the size of the circle is proportional to the relative frequency of the MC, the color around each circle indicates the lineage assigned to the MC, and the surface area of the colors inside the circles is proportional to the median fluorescence intensity (MFI) of canonical surface markers expressed on each MC (expression patterns of all surface markers in Supplemental Figure 3). NK = natural killer; TCR = T cell receptor.


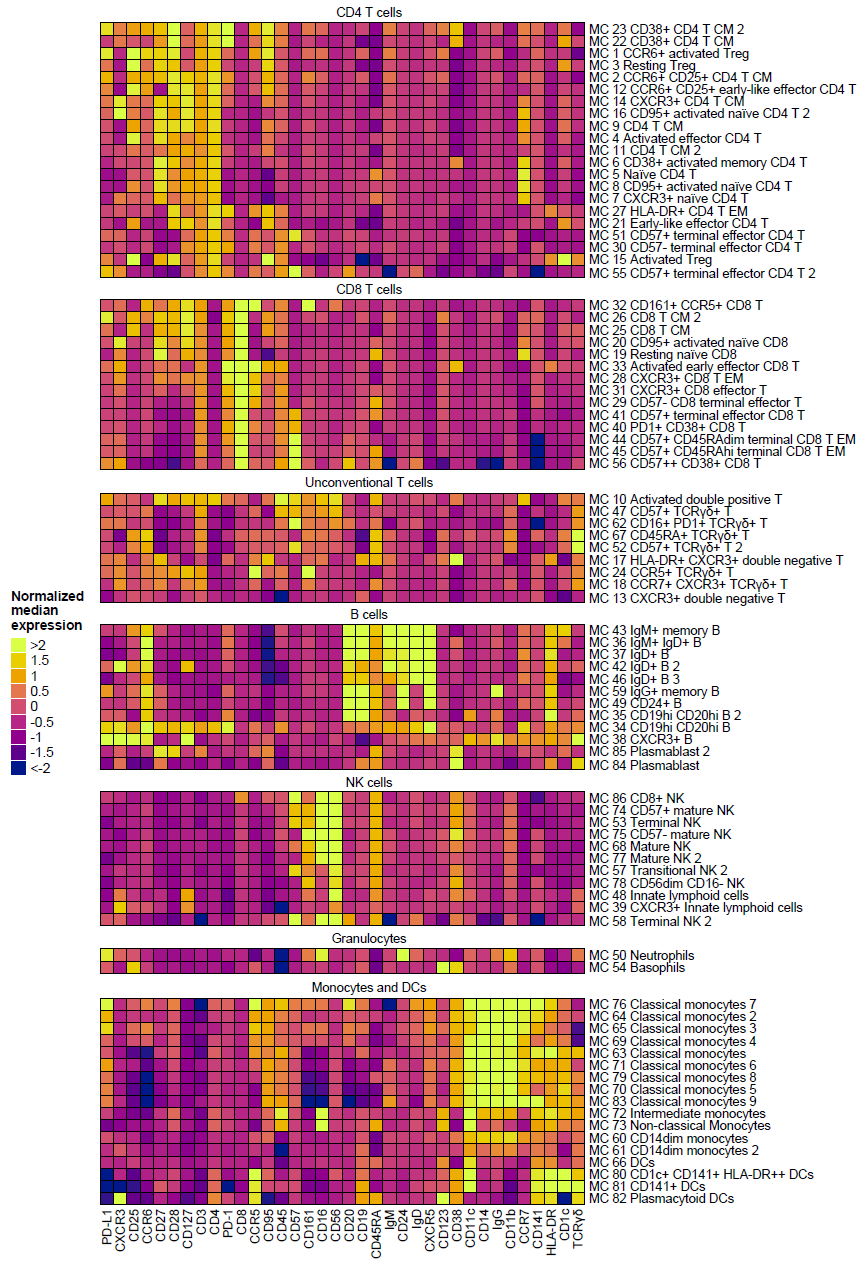


### Supplemental Figure 3

Heatmap depicting the normalized median fluorescence intensity (MFI) for all 36 surface markers on all 86 metaclusters (MCs), stratified by lineage. MFIs were normalized (i.e. transformed to a Z-score) per marker using the distribution of all MCs. CM = central memory; DC = dendritic cell; EM = effector memory; HLA-DR = human leukocyte antigen – DR isotype; NK = natural killer; PD-(L)1 = programmed death (ligand) 1; Treg = regulatory T cell.


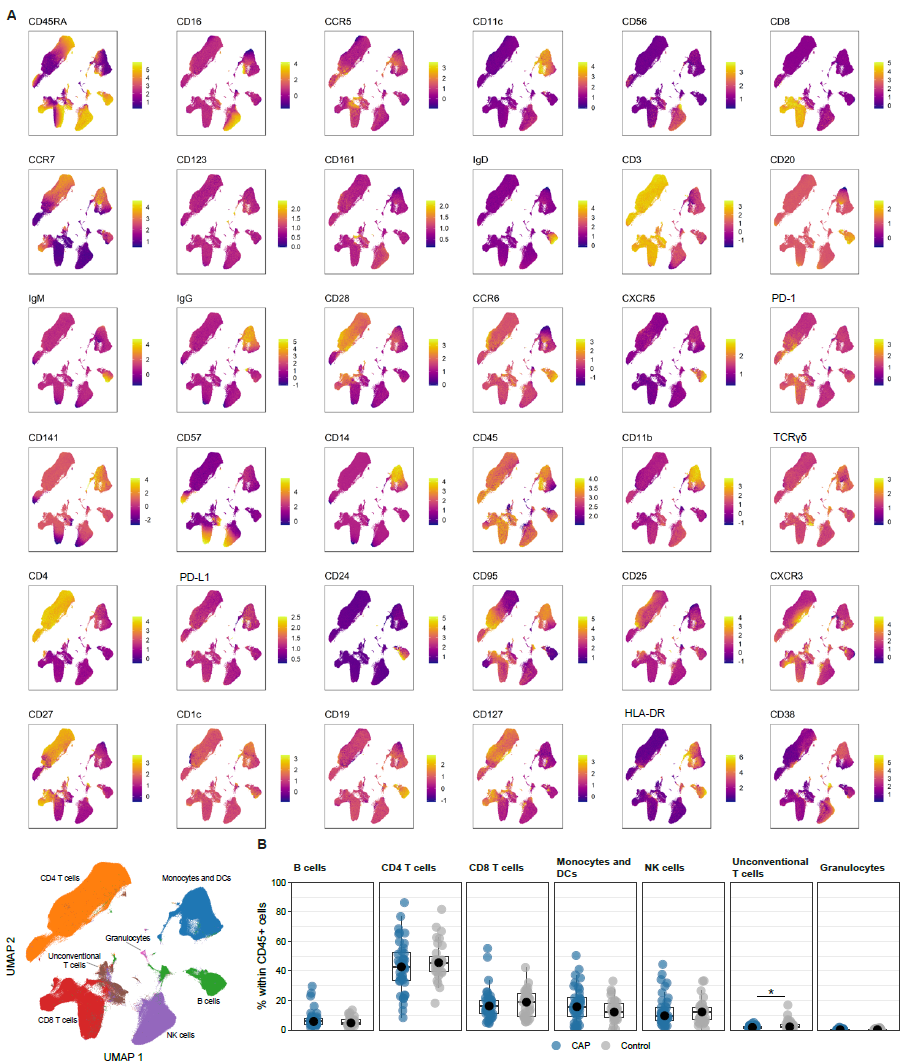


### Supplemental Figure 4

(*A*) Uniform manifold approximation and projection (UMAP) figures representative of all PBMCs from patients with CAP and controls (with Figure 1B displayed in the bottom left for reference), stratified per each of the 36 surface markers and colored by the expression level (fluorescence intensity) per cell. (*B*) Frequencies of the lineages as proportion of total CD45+ live cells per patient. Each colored dot represents an individual subject, the box represents the lower and upper quartile, the middle line and black dot represent the median. **P* <0.05. DC = dendritic cell; HLA-DR = human leukocyte antigen – DR isotype; NK = natural killer; PD-(L)1 = programmed death (ligand) 1.

###
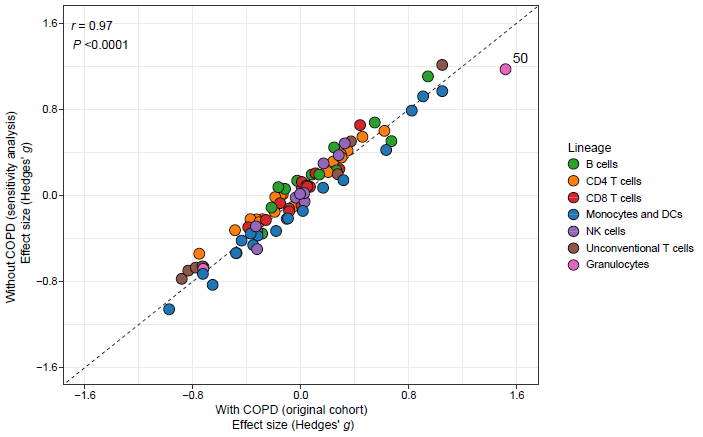
Supplemental Figure 5

The X-axis depicts the difference in the abundance, expressed as a Hedges’ *g* effect size, of all metaclusters (MCs) between all patients with CAP (n = 40) compared with all control subjects (n = 31). The Y-axis depicts the difference in the abundance of all MCs between patients with CAP without chronic obstructive pulmonary disease (COPD; n = 25) compared with the control subjects without COPD (n = 29). The closer a circle is to the dashed diagonal line with a slope of 1, the closer the effect sizes for the full cohort are to the cohort without COPD. The correlation between the effect sizes for all MCs is displayed as Pearson’s *r* in the top left corner. MC 50 (neutrophils) is highlighted because it is the only MC that is significant in a direct comparison between patients with CAP without COPD (n = 25) with patients with CAP with COPD (n = 15), *t*-test *P*-value = 0.003, but this comparison is no longer significant after adjusting the false discovery rate using the Benjamini-Hochberg method (adjusted *P* = 0.26). DC = dendritic cell; NK = natural killer.


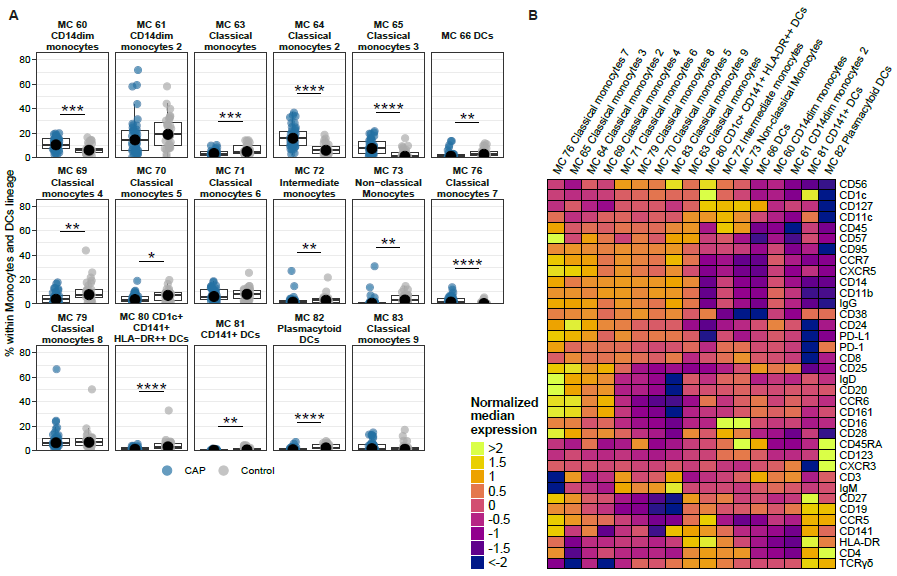


### Supplemental Figure 6

(*A*) Frequencies of monocyte and DC metaclusters (MCs) as proportions *within* total monocyte/DC cells per subject. Statistical comparisons between CAP and controls performed using a Wilcoxon rank-sum and not corrected for multiple testing. Each colored dot represents an individual subject, the box represents the lower and upper quartile, the middle line and black dot represent the median. **P* <0.05, ***P* <0.01, ****P* <0.001, *****P* <0.0001. (*B*) Heatmap depicting the normalized median fluorescence intensity (MFI) for all 36 surface markers on all monocyte/dendritic cell (DC) metaclusters MCs. MFIs were normalized (i.e. transformed to a Z-score) per marker using the distribution of all monocyte/DC MCs (to enhance the contrasts in marker expression when compared with Supplemental Figure 2). HLA-DR = human leukocyte antigen – DR isotype; PD-(L)1 = programmed death (ligand) 1.


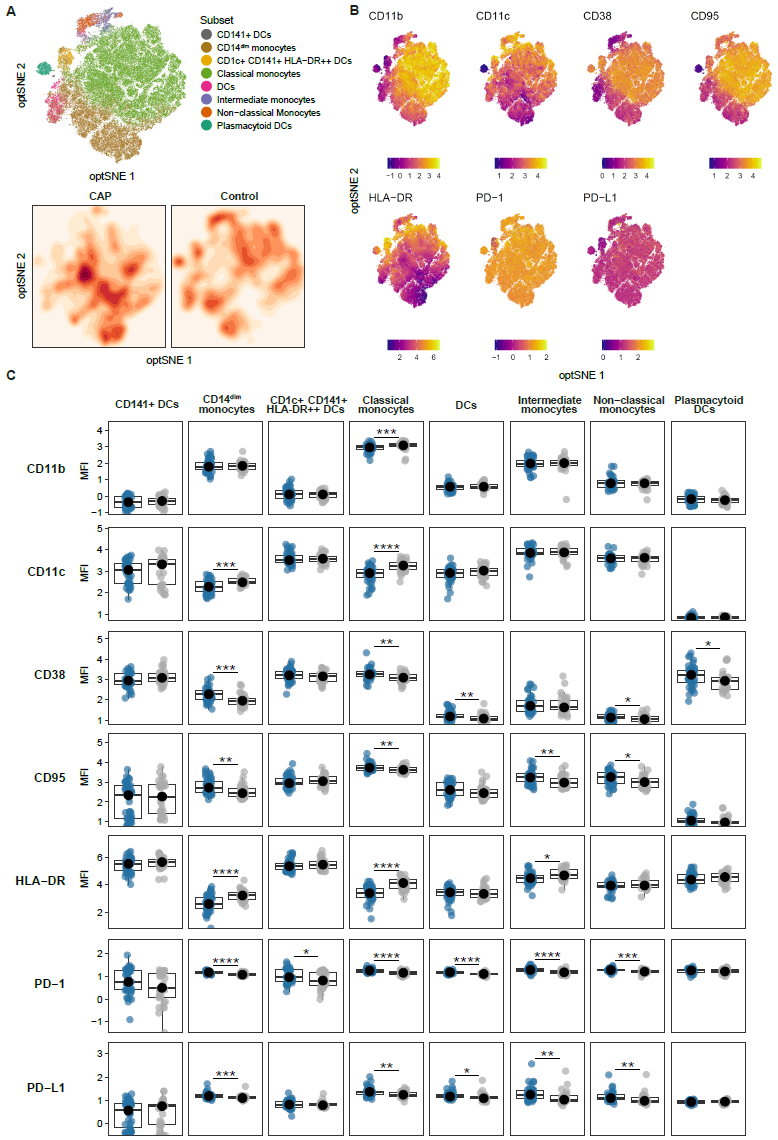


### Supplemental Figure 7

(*A*) Optimized t-distributed stochastic neighbor embedding (opt-SNE) plot representative of all monocytes and dendritic cells (DCs), colored by cell subset. The same plot is also depicted as density plot for both patients with CAP and control subjects. Darker red colors indicate a higher density of cells. (*B*) The same opt-SNE plots, colored by the expression level (fluorescence intensity) of the indicated surface markers per cell. (*C*) Boxplots showing the (arcsinh transformed) median fluorescence intensity (MFI) of the indicated markers for each subject within all subsets in the monocyte/DC lineage. Each colored dot represents an individual subject, the box represents the lower and upper quartile, the middle line and black dot represent the median. **P* <0.05, ***P* <0.01, ****P* <0.001, *****P* <0.0001. HLA-DR = human leukocyte antigen – DR isotype; PD-(L)1 = programmed death (ligand) 1.


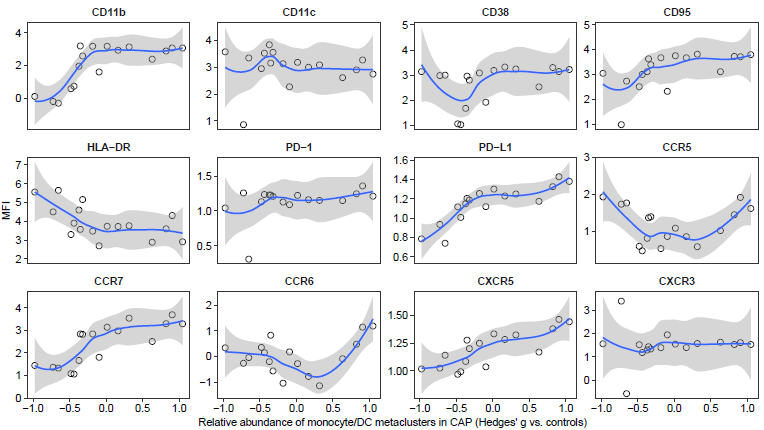


### Supplemental Figure 8

Exploratory scatterplots for the relationship between the expression of selected markers relevant for immune activation, suppression and migration on monocyte/dendritic cell (DC) metaclusters (MCs), and the relative abundance of monocyte/DC MCs. Y-axis shows (arcsinh transformed) median fluorescence intensity (MFI); X-axis shows the Hedges’ *g* effect size (positive means higher in CAP versus controls, negative means lower in CAP versus controls). The blue local regression line was fitted using the locally estimated scatterplot smoothing (LOESS) method. HLA-DR = human leukocyte antigen – DR isotype; PD-(L)1 = programmed death (ligand) 1.


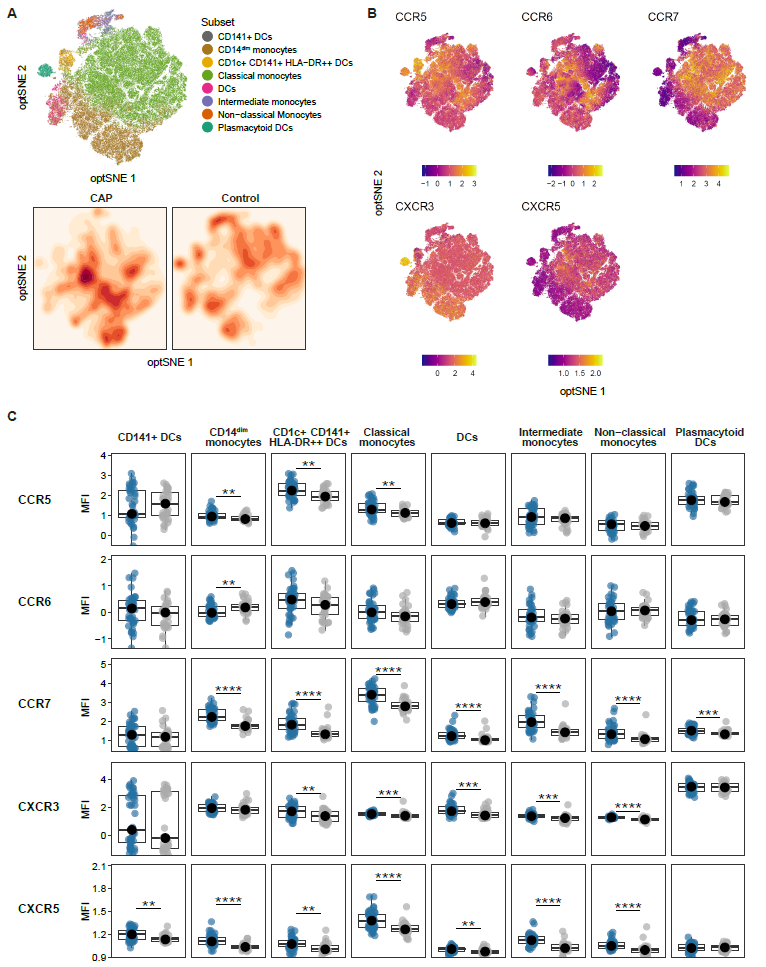


### Supplemental Figure 9

(*A*) Optimized t-distributed stochastic neighbor embedding (opt-SNE) plot representative of all monocytes and dendritic cells (DCs), colored by cell subset. The same plot is also depicted as density plot for both patients with CAP and control subjects. Darker red colors indicate a higher density of cells. (*B*) The same opt-SNE plots, colored by the expression level (fluorescence intensity) of the indicated surface markers per cell. (*C*) Boxplots showing the (arcsinh transformed) median fluorescence intensity (MFI) of the indicated markers for each subject within all subsets in the monocyte/DC lineage. Each colored dot represents an individual subject, the box represents the lower and upper quartile, the middle line and black dot represent the median. **P* <0.05, ***P* <0.01, ****P* <0.001, *****P* <0.0001. HLA-DR = human leukocyte antigen – DR isotype.


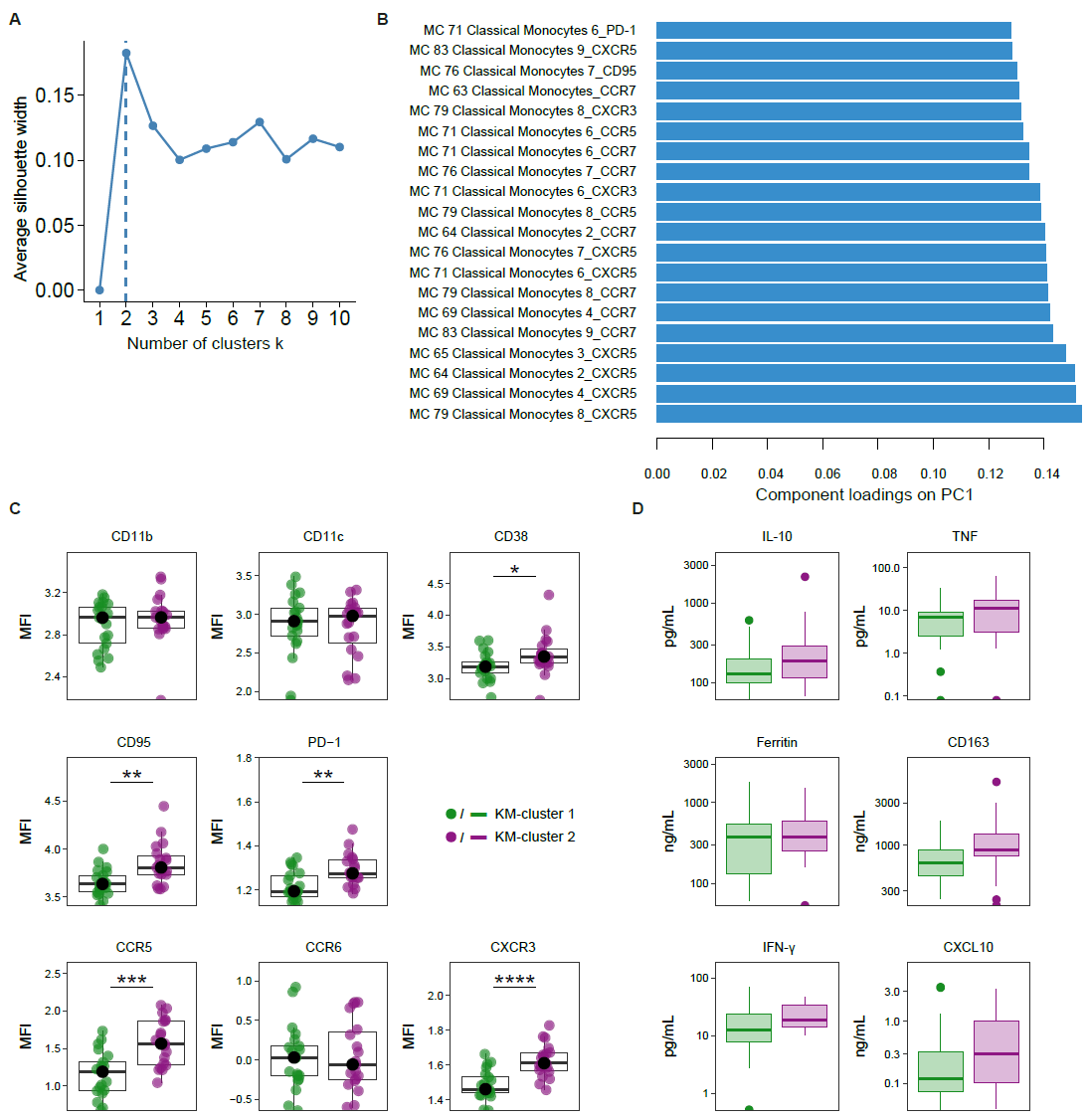


### Supplemental Figure 10

(*A*) Average silhouette width for *k* number of clusters for the K-means (KM) clustering analysis using surface markers on classical monocyte metaclusters (MCs). (*B*) The top 20 component loadings on principal component 1 (PC1) for the biplot depicted in Figure 4A. (*C*) Boxplots showing the (arcsinh transformed) median fluorescence intensity (MFI) of depicted surface markers for each subject within the classical monocyte subset, stratified by the two KM-clusters. Each colored dot represents an individual subject, the box represents the lower and upper quartile, the middle line and black dot represent the median. **P* <0.05, ***P* <0.01, ****P* <0.001, *****P* <0.0001. (*D*) Boxplots for the plasma concentrations of selected biomarkers indicative of inflammatory status in patients with available plasma samples in KM-cluster 1 (n = 20) and KM-cluster 2 (n = 17). The box represents the lower and upper quartile, the middle line represent the median, the whiskers represent the distribution up to 1.5 times the interquartile range beyond the lower or upper quartile, outliers are depicted as individual dots. HLA-DR = human leukocyte antigen – DR isotype; IFN-γ = interferon γ; PD-1 = programmed death 1; TNF = tumor necrosis factor.


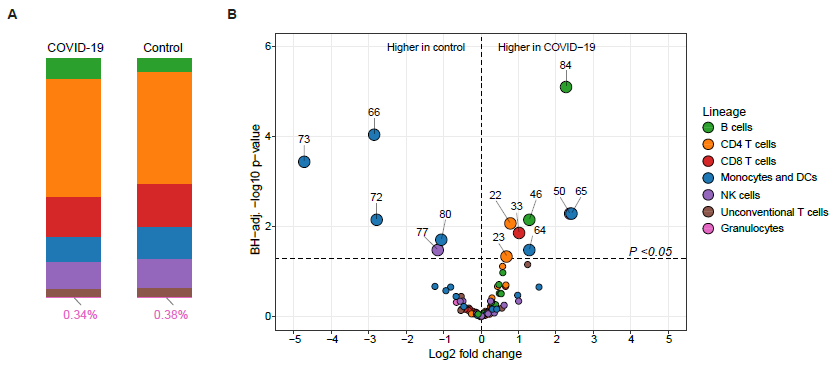


### Supplemental Figure 11

(*A*) Stacked bar charts indicating the mean of each lineage as a proportion of total cells for patients with COVID-19 and controls. The proportion of (contaminating) granulocytes in the PBMC fraction was very low and therefore indicated in text at the bottom of each bar. (*B*) Volcano plot for the comparison of all 86 metaclusters (MCs; as proportions of total number of cells per subject) between patients with COVID-19 and controls. The X-axis depicts the difference in means of the log2-transformed proportion of each MC, the Y-axis depicts the -log10-transformed Benjamini-Hochberg- (BH)-adjusted *P­­*-value obtained using Welch’s *t*-test. Larger labelled points above the horizontal line represent significantly differentially abundant MCs. DC = dendritic cell; NK = natural killer.
